# A Validated HPLC-MS/MS Assay for 14-*O*-[(4,6-Diaminopyrimidine-2-yl)thioacetyl] Mutilin in Biological Samples and Its Pharmacokinetic, Distribution and Excretion via Urine and Feces in Rats

**DOI:** 10.3390/molecules24040790

**Published:** 2019-02-22

**Authors:** Yunxing Fu, Yu Liu, Yunpeng Yi, Jianping Liang, Qingfeng Wu, Ruofeng Shang

**Affiliations:** 1 Key Laboratory of New Animal Drug Project of Gansu Province, Key Laboratory of Veterinary Pharmaceutical Development, Ministry of Agriculture, Lanzhou Institute of Husbandry and Pharmaceutical Sciences of CAAS, Lanzhou 730050, China; fyx1261648623@163.com (Y.F.); yangguang8684@163.com (Y.L.); yiyp@foxmail.com (Y.Y.); liangjp100@sina.com (J.L.); 2 Institute of Modern Physics, Chinese Academy of Sciences, Lanzhou 730000, China

**Keywords:** DPTM, HPLC-MS/MS, pharmacokinetics, distribution, excretion

## Abstract

14-*O*-[(4,6-Diaminopyrimidine-2-yl)thioacetyl] mutilin (DPTM), a novel pleuromutilin candidate with a substituted pyrimidine moiety, has been confirmed to possess excellent antibacterial activity against Gram-positive bacteria. To illustrate the pharmacokinetic profile after intravenous (i.v.), intramuscular (i.m.) and oral (p.o.) administrations with DPTM, as well as tissue distribution and excretion via urine and feces in vivo, a specific, sensitive and robust HPLC-MS/MS method was first developed to determine DPTM in rat plasma, various tissues, urine and feces. The plasma, tissues, urine and feces samples were treated by protein precipitation with acetonitrile using tiamulin fumarate as an internal standard (IS). This method which was achieved on an HPLC system detector equipped with an ESI interface, was sensitive with 5 ng/mL as the lower limit of detection and exhibited good linearity (R^2^ > 0.9900) in the range of 5–4000 ng/mL for plasma, various tissues, urine and feces, as well as intra-day precision, inter-day precision and accuracy. The matrix effects ranged from 94.2 to 109.7% with RSD ≤ 9.4% and the mean extraction recoveries ranged from 95.4 to 109.5% in plasma, tissue homogenates, urine and feces (RSD ≤ 9.9). After i.v., i.m. and p.o. administrations, DPTM was rapidly absorbed and metabolized in rats with the half-life (*t*_1/2_) of 1.70–1.86, 3.23–3.49 and 4.38–4.70 for 10, 25 and 75 mg/kg doses, respectively. The tissue distribution showed that DPTM was diffused into all the tested tissues, especially into the intestine and lung. Excretion via urine and feces studies demonstrated that DPTM was mainly excreted by feces after administration.

## 1. Introduction

Pleuromutilin was first discovered and isolated in a crystalline form from cultures of *Pleurotus mutilus* and *P. passeckerianus* in 1951 [1,2,3,4,5]. Pleuromutilin and its derivatives selectively inhibit bacterial protein synthesis by interacting with the peptidyl transferase centre (PTC) of prokaryotic ribosomes at the A- and P-site [6,7,8]. Further studies using a crystal structure of *Deinococcus radiodurans* 50S ribosomal subunit complex with tiamulin revealed that the tricyclic core of the tiamulin are mediated through hydrophobic interactions and hydrogen bond at the A-site, while the C-14 side chain pointing toward the P-site at the PTC [9,10,11]. 

Modifications of the pleuromutilin side chain may improve its biological activities and thus led to the discovery of three drugs: tiamulin [12], valnemulin [13,14], and retapamulin [15,16]. The subsequent pleuromutilin drug: lefamulin (BC-3781) has entered Phase III clinical trials and received US FDA fast-track status to treat community-acquired bacterial pneumonia (CABP) and acute bacterial skin and skin structure infections (ABSSSI) [17,18,19,20]. 

**Figure 1:**
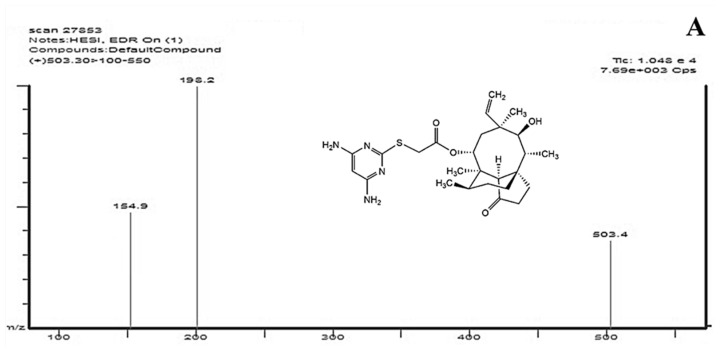

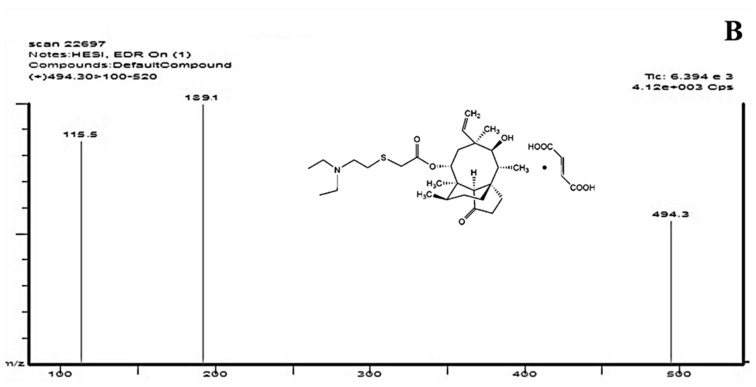
Structures and mass spectra of DPTM (**A**) and tiamulin fumarate (**B**).

In our previous work, we synthesized a novel pleuromutilin derivative: 14-*O*-[(4,6-Diaminopyrimidine-2-yl)thioacetyl] mutilin (DPTM, Figure 1A). This compound displayed excellent in vitro activities against Gram-positive bacteria, including methicillin-resistant *Staphylococcus aureus* (MRSA), *Staphylococcus aureus* (*S. aureus*), *Bacillus subtilis* (*B. subtilis*) [21]. These significant antibacterial activities provide the basis for the further in-depth drug targeted studies of DPTM as a novel antibacterial agent. 

Preclinical pharmacokinetic studies play an important role that can be used to make critical decisions supporting the safety and efficacy in the development of new drugs [22]. To our knowledge, there is no report on the pharmacokinetics, tissue distribution and excretion via urine and feces studies of DPTM, as well as the determination of DPTM in rat plasma, various tissues, urine and feces using UPLC-based method. However, a few literatures were reported on the determination and pharmacokinetic study of other pleuromutilin derivatives, such as lefamulin [20], valnemulin [23] tiamulin [24] and 14-*O*-[(2-amino-1,3,4-thiadiazol-5-yl) thioacetyl] mutilin [25]. In the present study, an HPLC-MS/MS approach was developed and validated for the first time for analyzing DPTM in rat plasma, various tissues, urine and feces. Using this method, we conducted the preclinical pharmacokinetics, excretion via urine and feces and tissue distribution properties of DPTM in rats after intravenous (i.v.), intramuscular (i.m.) and oral (p.o.) administrations, respectively. 

## 2. Results and Discussion

### 2.1. Optimization of HPLC-MS/MS Conditions

We chose positive ion monitoring mode after infusing DPTM (640 ng/mL) and tiamulin fumarate (540 ng/mL, Figure 1B) used as internal standard (IS) into the mass spectrometer in both positive and negative mode. Because of the two amino groups in DPTM, the collision behavior of [M + H]^+^ is strongly dependent on the collision energy (CE), which was determined by observing the response of the obtained fragment ion peaks. Two major fragment ions for DPTM at *m*/*z* 198.2 and 151.4 were formed when the CE at 11 and 30 eV, respectively. For IS the two fragment ions at *m*/*z* 189.1 and 115.1 were formed when the CE at 19 and 37 eV, respectively. Therefore, the MRM transitions were monitored at *m*/*z* 503.4 → 198.2 and 503.4 → 151.4 for DPTM (Figure 1A) and at *m*/*z* 494.3 → 189.1 and 494.3 → 115.5 for IS (Figure 1B) at 4 kV spray voltage. 

To obtain the best MS response and optimal chromatographic behavior, an isocratic elution system of different ratio of the organic phase (methanol and acetonitrile) and water phase were investigated. We chose 50% acetonitrile and 50% water (0.1% formic acid) as the mobile phase because of the better peak shape and lower background noise for the analyte. 

### 2.2. Method Validation

#### 2.2.1. Specificity

**Figure 2:**
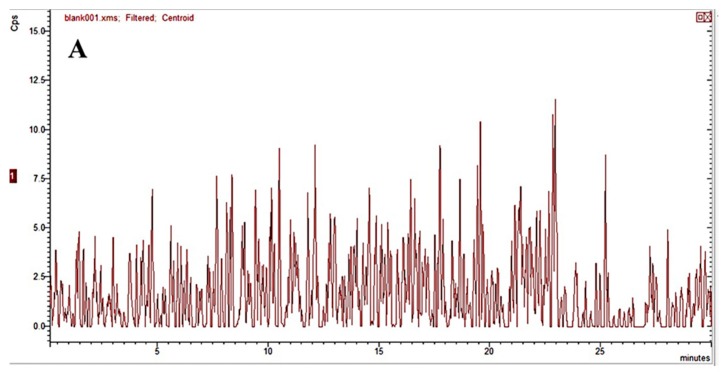

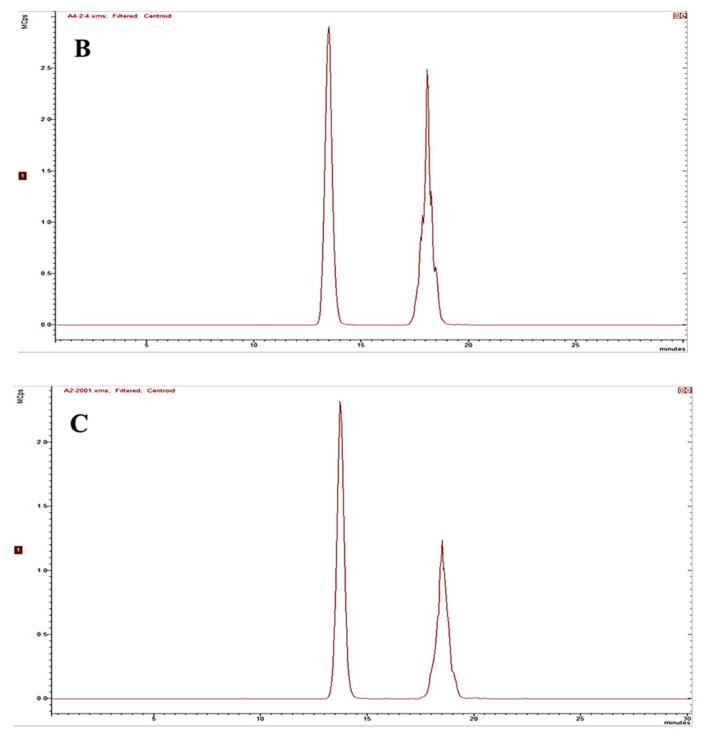
Typical chromatograms of blank rat plasma (**A**), blank rat plasma spiked with DPTM and IS (**B**), plasma sample 1 h after i.v. administration (**C**) of DPTM. The retention times of DPTM and IS were 13.8 and 18.7 min, respectively.

The specificity was assessed by analyzing whether there were interferences with blank biological samples (blank plasma, tissue homogenates, urine and feces), blank biological samples spiked with DPTM and IS, and the biological samples after administration of DPTM. The typical DPTM chromatograms of blank rat plasma, blank rat plasma spiked with DPTM and IS, and plasma sample from a rat 1 h after i.v. administration are shown in Figure 2. Typical DPTM chromatograms of blank biological samples (except for plasma) were reported in Supplemental Data. Under the developed HPLC-MS/MS conditions, no significant endogenous interference was observed at the retention time of DPTM (13.8 min) and IS (18.7 min). 

#### 2.2.2. Linearity and Sensitivity

The calibration curve was acquired by plotting the ratio of peak areas of DPTM to that of IS against the nominal concentration of calibration standards. The regression equations were weighted with a factor of 1/x. 

The calibration curves for DPTM in plasma, tissue homogenates, urine and feces were linear in the concentration range of 5–4000 ng/mL. Typical calibration curves, correlation coefficients and linear ranges for DPTM in plasma, tissue homogenates, urine and feces are listed in Table 1. The higher correlation coefficients (R^2^ > 0.9900) of the calibration curves showed good linearity over the wide concentration ranges. The lower limit of the quantification (LLOQ) of the assay was determined by assessing the lowest concentration in the standard curve that could be quantified with 80–120% accuracy and precision (variation coefficient < 20%). The LLOQ values for DPTM in all samples was 5 ng/mL. 

#### 2.2.3. Accuracy and Precision

The intra- and inter-day accuracy (% RE) and precision (% RSD) were determined by analyzing three levels of quality control samples (QCs) (10, 200 and 3000 ng/mL) on three different days with six replicates at each concentration per day (Table 2). 

The intra- and inter-day accuracy for blank plasma, tissue homogenates, urine and feces were ≤9.8% and ≤9.7%, respectively, and the precision were ≤9.8% and ≤9.5%, respectively. The results indicate that the present method has good accuracy and precision. 

#### 2.2.4. Extraction Recovery and Matrix Effects

The extraction recovery was determined by comparing the peak areas of six extracted plasma, tissue homogenates, urine and feces samples at three QC concentrations (10, 200 and 3000 ng/mL) with those of spiked–after–extraction samples. The mean extraction recovery of DPTM at three levels of QC and IS samples were ranged from 95.4 to 109.5% in plasma, tissue homogenates, urine and feces (RSD ≤ 9.9). The results indicated that the extraction procedure was acceptable. Matrix effects were calculated according to the formula: Peak area of blank sample extracts spiked with the analyte / Peak area of pure standard solution containing equivalent amounts of analyte × 100%. The recovery and the matrix effect of IS were determined in the same way. The absolute matrix effect values were in the range of 94.2–109.7%, which indicated no obvious matrix effect for DPTM in biological samples (Table 3). 

#### 2.2.5. Dilution Integrity

The dilution integrity of DPTM was investigated by analyzing six replicate samples with the concentration of 80,000 ng/mL which were diluted 20-fold with blank rat matrix. These diluted samples were analyzed using freshly prepared calibration curve to calculate RE and RSD with 7.6% and 9.3%, respectively, which were within the acceptable limit of 15%. This result demonstrated that the developed method could be applied for the higher concentrations exceeding the linear ranges during quantitative analysis. 

#### 2.2.6. Stability

Stability of DPTM and IS was examined by comparing the analytes’ concentrations in freshly prepared samples with those of samples maintained under different conditions, including the 24 h storage at room temperature, three freeze and thaw cycles, and storage at −70 °C for 4 weeks, respectively. Analyzing three levels of QC samples were used to assess the stability (accuracy and precision) with six replicates. The results of stability experiments (% RE and % RSD) were all in 15%, which indicated that the plasma, tissue homogenates, urine and feces samples are stable under the above conditions (Table 4). 

### 2.3. Pharmacokinetic Profiles

**Figure 3:**
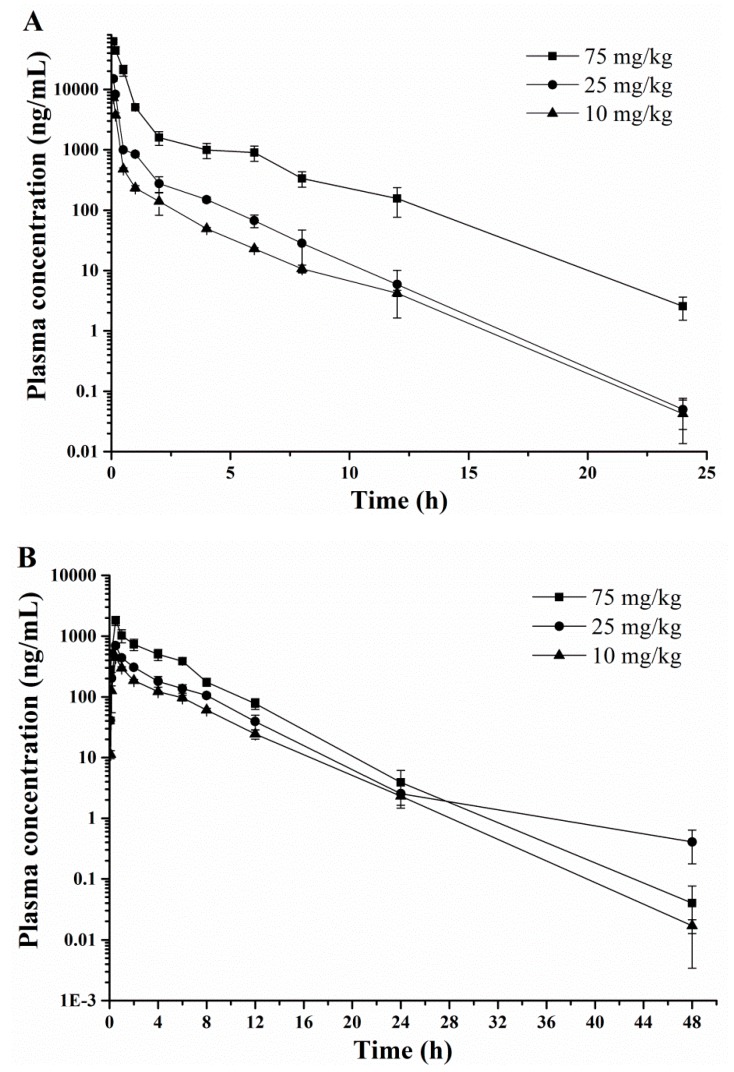

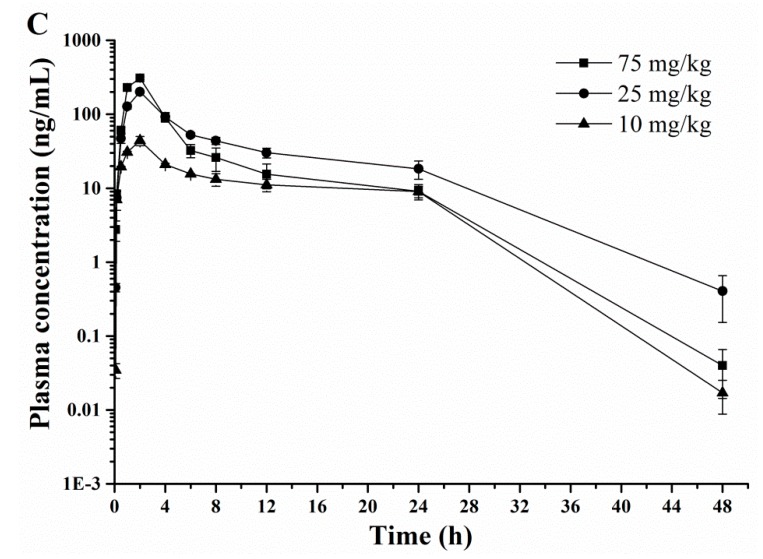
Plasma concentration-time curve of DPTM after i.v. (**A**), i.m. (**B**) and p.o. (**C**) administration to rats.

After administration of three i.v., i.m. and p.o. dose (10, 25 and 75 mg/kg) of DPTM to rats, the pharmacokinetic profiles of DPTM were quantified by the validated HPLC-MS/MS methods. Estimated non-compartmental parameters of DPTM are presented in Table 5. The mean concentration-time curves in plasma of DPTM after i.v., i.m. and p.o. administration are shown in Figure 3. In our study, the dose-dependent maximum concentration (*C*_max_) of drug in serum were 7.54–65.10, 0.47–1.83 and 0.05–0.31 μg/mL for 10, 25 and 75 mg/kg doses, which were observed at 0.08, 0.50 and 2.00 h after i.v., i.m. and p.o. administration of DPTM, respectively. This reflected a rapidly absorption within 2 h, similar to that of other pleuromutilin derivatives, for example, 14-*O*-[(2-amino-1,3,4-thiadiazole-5-yl)thioacetyl] mutilin [26] which *C*_max_ was achieved at 0.08 and 0.75 h in rats after i.v. and p.o. administration (5 mg/kg dose), and 14-*O*-[(4-amino-6-hydroxy-pyrimidine-2-yl) thioacetyl] mutilin with *C*_max_ achieved at 0.08 h in rabbits after i.v. administration (25 mg/kg dose) [27]. The half-life (*t*_1/2_) was 1.70–1.86, 3.23–3.49 and 4.38–4.70 h for three different doses. In addition, the area under the curve from 0 to 48 h (AUC_0→t_) was 2.23–35.98, 1.52–5.70 and 0.51–1.50 μg·h/mL for 10, 25 and 75 mg/kg doses after i.v., i.m. and p.o. administration, respectively, suggesting dose-dependency in the pharmacokinetics. The absolute bioavailabilities of DPTM were 68.16%, 48.52% and 15.84% and 22.87%, 24.26%, and 4.17% following i.m. and p.o. dose administration in rats, respectively. These results indicated the absolute bioavailabilities following oral administration were lower than that of 14-*O*-[(2-amino-1,3,4-thiadiazole-5-yl)-thioacetyl] mutilin in rats (35.81%) [26], valnemulin (a pleuromutilin drug used for animals) in ducks (36.68%) and in rats (100%) [23]. 

### 2.4. Tissue Distribution

**Figure 4:**
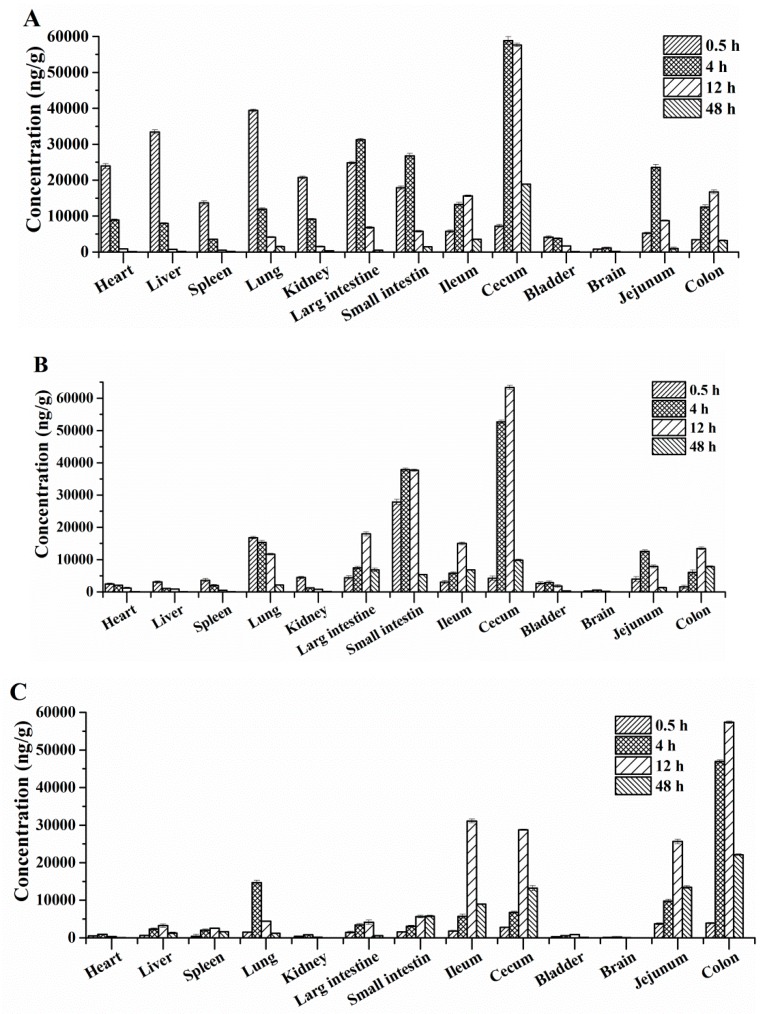
Mean concentration of DPTM in various tissues at 0.5, 4, 12 and 48 h after i.v. (**A**), i.m. (**B**) and p.o. (**C**) administration at a dose of 75 mg/kg (*n* = 12, mean ± SD).

We also successfully used the established LC-MS/MS method for detection of the concentration of DPTM in rat tissue homogenates. The quantification of DPTM used the specific standard curves in each corresponding bio-matrix. After i.v., i.m. and p.o. administration of 75 mg/kg DPTM, tissue distribution was investigated at 0.5, 4, 12 and 48 h by collecting the heart, liver, brain, spleen, lung, kidney, large intestine, small intestine, ileum, cecum, bladder, jejunum and colon. The concentration-time profiles of DPTM in various tissues are shown in Figure 4. 

We observed that DPTM underwent a wide distribution into all tested tissues, especially mainly into the intestine and lung. The highest levels of DPTM were observed in jejunum (23543 ± 799, 12551 ± 432 and 38769 ± 546 ng/mg), cecum (62457 ± 675, 63404 ± 407 and 31087 ± 452 ng/mg), large intestine (31241 ± 355, 17978 ± 116 and 4123 ± 567 ng/mg) small intestine (26753 ± 690, 37890 ± 437 and 6743 ± 474 ng/mg) and lung (39411 ± 357, 15345 ± 579 and 14685 ± 654 ng/mg) within the first 24 h after i.v., i.m. and p.o. administration, respectively. The concentration of DPTM in all tested tissues increased within the first 12 h, and then decreased in the following 24 h. However, the colon had a large amount of DPTM until 48 h (3214 ± 177, 7849 ± 243 and 22114 ± 215 ng/mg after i.v., i.m. and p.o. administration, respectively). Perhaps this compound can be developed into a new drug used to treat intestinal infections. 

### 2.5. Excretion via Urine and Feces

**Figure 5:**
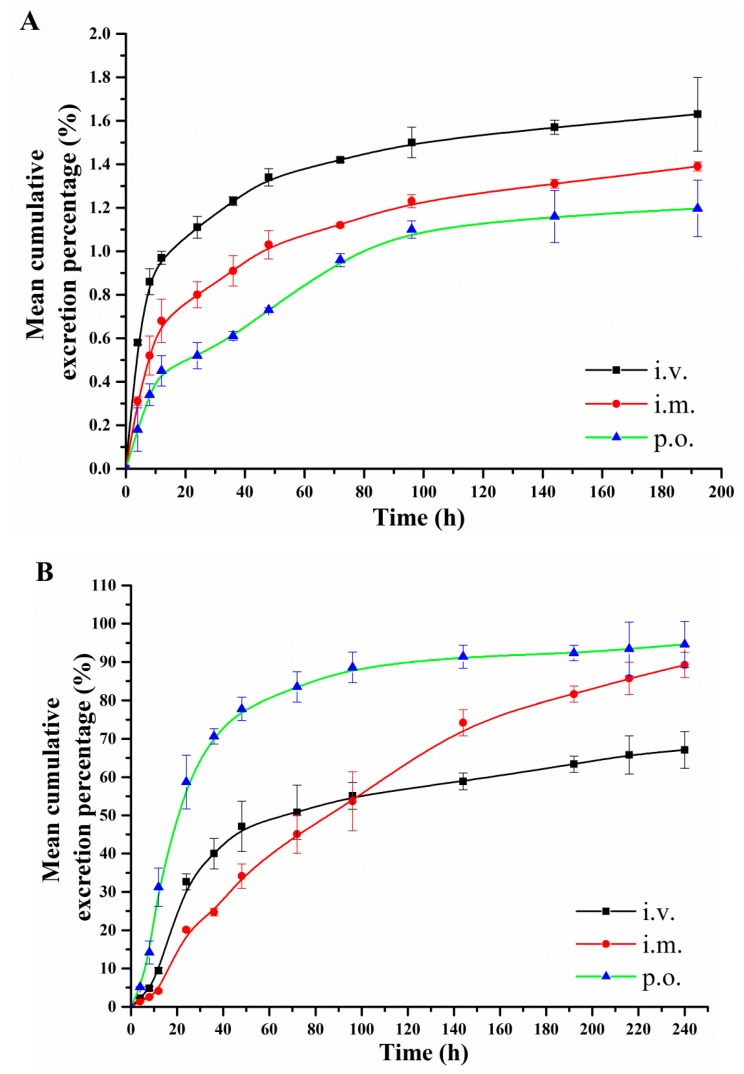
Mean cumulative excretion percentage-time profiles of DPTM in rat urine (**A**) and feces (**B**) following i.v., i.m. and p.o. administration at a dose of 75 mg/kg (*n* = 6).

The cumulative excretion of DPTM in rat urine (within 192 h) and feces (within 240 h) after a single i.v., i.m. and p.o. administrationof 75 mg/kg, respectively, are illustrated in Figure 5. 

The results showed that DPTM was rapidly excreted at 48 h post-treatment and then slowly from 48 h to 192 h (urine) or 240 h (feces) post-treatment. The total prototype drug was detected mainly in feces with 47.13%, 34.11% and 77.78% after i.v., i.m. and p.o. administration, respectively, which demonstrate that DPTM were excreted mainly via feces after administration. Furthermore, the prototype drug was still excreted with 1.63%, 1.39% and 1.20% in urine at 192 h and 67.09%, 89.18% and 94.62% in feces at 240 h, respectively. However, at the last acquisition time point, DPTM in urine (192 h) and feces (240 h) was lower than that of the LLOQ. 

## 3. Materials and Methods

### 3.1. Chemicals and Reagents

DPTM was synthesized in our laboratory using the standard procedure [21]. The purity of this compound was checked by HPLC and quantitative NMR analyses to be 98.72%, and the structure of synthetic DPTM was confirmed by IR, NMR and HR-MS spectrometry. Tiamulin fumarate (purity of 98.5%) was purchased from Dr. Ehrenstorfer GmbH (Augsburg, Germany). Acetonitrile, methyl alcohol and formic acid purchased from Fisher Scientific (Fair Lawn, NJ, USA) were of HPLC grade. Ultrapure water was purchased from A.S. Watson Group (Guangzhou) Ltd. (Guangzhou, China) and used throughout the study. All the other chemicals used were of analytical grade and obtained from commercial sources. 

### 3.2. Animals

One hundred and twenty Sprague-Dawley rats (270 ± 20 g of 6–8 weeks old, half male and half female) were purchased from Laboratory Animal Center of Lanzhou University (Lanzhou, China). The animals were maintained under 23 °C with a constant 12 h light-dark cycle and free access to food and water. The experimental protocol, approved by the Committee for Ethics in Laboratory Animal Center of Lanzhou University (number: SCXK2013-0002), was conducted in strict accordance with the Ethical Principles in Animal Research. 

### 3.3. HPLC and Mass Spectrometry

Chromatographic separation was carried out on an Advance HPLC system (Bruker Daltonics Inc., Billerica, MA USA) using isocratic elution. An ZORBAX SB-C18 column (Agilent, Santa Clara, CA, USA, 4.6 mm × 250 mm, 5 μm) was employed and the temperature of column oven was set at 40 °C. The mobile phase consisted of acetonitrile-water with 0.1% formic acid (50:50, *v*/*v*). The auto-sampler was conditioned at 6 °C and the injection volume was 10 μL for analysis. 

Triple-quadrupole tandem mass spectrometric detection was carried out on a Bruker EVOQ Qube (Bruker Daltonics Inc.) operated in the positive ion mode. Quantification of DPTM was performed with multiple reactions monitoring (MRM), and the transitions were set at *m*/*z* 503.4 → 198.2 (collision energy 11 eV, argon collision gas pressure 1.5 m Torr, scan time 0.5 s) and *m*/*z* 503.4 → 151.4 (collision energy 30 eV, argon collision gas pressure 1.5 m Torr, scan time 0.5 s), respectively. For IS, the transitions were set at *m*/*z* 494.3 → 189.1 (collision energy 19 eV, argon collision gas pressure 1.5 m Torr, scan time 0.5 s) and *m*/*z* 494.3 → 115.5 (collision energy 37 eV, argon collision gas pressure 1.5 m Torr, scan time 0.5 s), respectively. The spray voltage was set to 4.0 kV and the source temperature was maintained at 350 °C. All data were acquired and integrated by MS Workstation V8.2 software.3.4 (Bruker Daltonics, Bremen, Germany). Standard and sample preparation. 

### 3.4. Preparation of Stock and Working Solutions

Stock solutions of DPTM (0.4 mg/mL) and IS (0.2 mg/mL) were separately prepared in acetonitrile and stored at 4 °C. Subsequently, the working standard solution of DPTM was prepared by diluting the stock solution in acetonitrile to obtain a linear concentration gradient: 0.05, 0.2, 0.5, 1, 2, 5, 10, 20 and 40 μg/mL. A 20 μg/mL working solution of IS was obtained by diluting the stock solution with acetonitrile. All working solutions were stored at 4 °C until analysis. 

### 3.5. Preparation of Calibration Standards and Quality Control (QC) Samples

For the construction of calibration curves, 20 μL of each corresponding standard working solution (0.05, 0.2, 0.5, 1, 2, 5, 10, 20 and 40 μg/mL) was evaporated to dryness by nitrogen at 40 °C. The calibration standards (5, 20, 50, 100, 200, 500, 1000, 2000 and 4000 ng/mL) were prepared by adding 200 μL of blank plasma, various tissue homogenates (including heart, liver, spleen, lung, kidney, large intestine, small intestine, ileum, cecum, bladder, brain, jejunum and colon) or urine and feces to the residue, respectively. Quality control (QC) samples (10, 200 and 3000 ng/mL) were obtained by the same way as the calibration standards. All solutions were stored at −70 °C before use for no longer than 4 weeks. 

### 3.6. Sample Treatment

Calibration standards, QC samples and all the bio-samples (plasma samples, vary tissue homogenates, urine and feces) were treated with protein precipitation method by acetonitrile. The procedure was as follows: 200 μL samples was mixed with 800 μL acetonitrile and then vortexed for 5 min and centrifuged at 12,000 rpm for 10 min. The supernatant was transferred into a clean tube, and evaporated with a stream of nitrogen. The residue was treated with the above mentioned procedure again. The final residue was stored at −70 °C. Before analysis, the residue was dissolved with certain volume mobile phase. 

### 3.7. Method Validation

The validation of DPTM determination in plasma, tissue homogenates, urine and feces was conducted in terms of specificity, linearity, sensitivity, accuracy, precision, extraction recovery, matrix effect, stability and dilution integrity according to the EMEA guidelines [28,29]. 

### 3.8. Pharmacokinetic Study

Single i.v., i.m. and p.o. administrations of DPTM (dissolved in 3.0% soybean lecithin solution) were given to six rats at dose levels of 75, 50 and 10 mg/kg, respectively. Blood samples (approximately 200 µL) were collected into heparinized tubes through an indwelling catheter inserted in the jugular vein [30] at 0.083, 0.166, 0.5, 1, 2, 4, 6, 8, 12 and 24 h after i.v. dosing and at 0.083, 0.166, 0.5, 1, 2, 4, 6, 8, 12, 24 and 48 h after i.m. or p.o. dosing. The samples were immediately centrifuged (4000 rpm for 10 min) and the supernatants were taken out and stored in a freezer at −70 °C until analysis. The drug was extracted with acetonitrile using the condition previously described. Then, an aliquot of the reconstituted extracts was transferred and filtrated through a 0.22-μm cellulose membrane filter and 10 μL was injected into the HPLC-MS/MS system. 

A non-compartmental model was used to determine the pharmacokinetic parameters of DPTM. Pharmacokinetic parameters, including maximum plasma concentration (*C*_max_), time (*T*_max_), area under the plasma concentration versus time curve from zero to last sampling time (AUC_0−t_) and elimination half-life (*t*_1/2_) were calculated using WinNonlin software (version 2.1, Pharsight, Mountain View, CA, USA). The absolute bioavailability was calculated as follows:

*F* = (AUC_extravascular_/(AUC_i.v._) × (dose_i.v._/dose_extravascular_) × 100%.

The data are presented as mean ± standard deviation (SD) except for *T*_max_ values using median (minimum value–maximum value). 

### 3.9. Tissue Distribution Study in Rats

Tissue distribution study in rats was performed as described previously [31]. Animals were divided into three groups (*n* = 12) and DPTM was administered via i.m., i.v. and p.o. with a single dose of 75 mg/kg. A series of heart, liver, spleen, lung, kidney, large intestine, small intestine, ileum, cecum, bladder, brain, jejunum and colon samples were collected before and at 0.5, 4, 12 and 48 h time points after administration and rinsed with phosphate buffer to remove the blood and feces, followed by weighing the wet weight and freezing at −70 °C until analysis. The concentrations of DPTM in rat various tissues were expressed in ng/g and calculated by equation: Ct = Cs × Vs/P, where Ct represented the tissue concentration (ng/g), Cs, Vs and P were the concentration (ng/mL), the volume (mL) and the weight (g) of the tissue samples, respectively. 

### 3.10. Excretion via Urine and Feces Study in Rats

Excretion via urine and feces was studied in another six rats (half male and half female). After the rats were administered via a single i.v., i.m. and p.o. dose (75 mg/kg) of DPTM, urine and feces samples were collected at 0–4, 4–8, 8–12, 12–24, 24–36, 36–48, 48–72, 72–96, 96–144, 144–192 and 192–240 h intervals in plastic centrifuge tubes and stored at −70 °C until analysis. For quantitative analysis, the fecal samples were dried at 45 °C for 24 h and prepared by the established sample treatment procedure. The urinary samples were prepared by the established sample treatment procedure directly. The obtained samples were injected into the HPLC-MS/MS system for analysis. 

## 4. Conclusions

In this study, we developed a rapid, sensitive and reproducible HPLC-MS/MS method for the quantification of DPTM in plasma, various tissues, urine and feces samples. The method was validated according to the EMEA guidelines for industry and was useful for investigating the pharmacokinetics, tissue distribution and excretion via urine and feces. In the pharmacokinetic study, DPTM showed dose-dependent pharmacokinetic changes and was found to be rapidly absorbed and have fast clearance. Absolute bioavailabilities were 68.16%, 48.52% and 15.84% and 22.87%, 24.26%, and 4.17% when administered three doses via i.m. and p.o. in rats, respectively. In the tissue distribution study, DPTM was distributed slowly and widely in various tissues, especially in intestine and lung. In addition, DPTM was rapidly excreted within 48 h and slowly thereafter, mainly through the feces. These results provide useful data and information for further development of DPTM as a potential clinical antibacterial candidate. 

## Figures and Tables

**Table 1:** Linearity curve of DPTM in plasma, tissue homogenates, urine and feces samples.

Biosamples	Y = a X + b	95% CI for Slope	95% CI for Ordinate	R^2^	Linear Range (ng/mL)	LLOQ (ng/mL)
Plasma	Y= 467.7 X + 46.8	397.5–537.9	42.6–51.0	0.9999	5–4000	5
Heart	Y = 525.3 X + 131.4	439.2–609.4	120.9–141.9	0.9968	5–4000	5
Liver	Y = 533.3 X + 50.4	465.0–601.6	43.2–57.6	0.9988	5–4000	5
Spleen	Y = 682.8 X + 95.9	589.3–776.3	274.9–342.1	0.9963	5–4000	5
Lung	Y = 715.6X + 308.5	609.4–821.8	42.3–68.7	0.9987	5–4000	5
Kidney	Y = 664.5 X + 55.5	574.7–754.6	47.2–63.8	0.9951	5–4000	5
Large intestine	Y = 893.0 X + 341.4	803.7–982.3	308.9–373.8	0.9923	5–4000	5
Small intestine	Y = 733.3 X +82.5	674.6–791.9	43.5–121.5	0.9975	5–4000	5
Ileum	Y = 620.8X + 86.0	574.2–667.4	66.7–105.3	0.9978	5–4000	5
Cecum	Y = 689.9 X + 249.2	638.2–741.6	207.5–290.9	0.9993	5–4000	5
Bladder	Y = 664.5 X + 55.5	604.7–724.3	41.9–69.1	0.9988	5–4000	5
Brain	Y = 599.2 X + 25.3	563.2–635.2	17.4–33.2	0.9965	5–4000	5
Jejunum	Y = 624.7X + 95.3	574.7–674.7	70.0–120.6	0.9985	5–4000	5
Colon	Y = 664.8 X + 182.5	614.9–714.7	150.2–214.8	0.9996	5–4000	5
Urinary	Y= 514.4 X + 35.6	468.1–560.7	28.1–43.1	0.9995	5–4000	5
Fecal	Y=430.4 X + 24.1	387.4–473.4	19.1–29.1	0.9995	5–4000	5

CI: Confidence interval; R^2^: correlation coefficient. LLOQ: low limits of quantifications.

**Table 2:** Intra-and inter-day precision and accuracy obtained for DPTM analysis in plasma, tissue, homogenates, urine and feces (*n* = 6).

Biosamples	Spiked Concentration (ng/mL)	Intra-Day		Inter-Day	
		Precision (% RSD)	Accuracy (% RE)	Precision (% RSD)	Accuracy (% RE)
Plasma	10	6.1	7.9	5.9	8.1
	200	3.2	4.6	3.1	4.3
	3000	2.1	3.8	1.9	3.5
Heart	10	7.9	8.4	7.7	8.1
	200	0.5	0.3	0.4	0.4
	3000	3.6	4.5	3.2	4.1
Liver	10	7.9	6.8	7.7	6.5
	200	3.5	5.7	3.3	6.1
	3000	2.5	−0.8	5.3	−0.9
Spleen	10	3.5	4.6	2.3	4.3
	200	3.6	3.8	3.7	3.5
	3000	2.6	5.6	3.5	5.8
Lung	10	5.4	3.8	5.1	3.6
	200	2.9	−1.6	3.7	−1.3
	3000	3.3	5.5	3.3	5.3
Kidney	10	7.5	9.8	7.7	8.8
	200	3.6	4.3	3.2	4.8
	3000	2.1	8.3	2.3	8.9
Large intestine	10	4.6	4.3	3.9	4.1
	200	9.8	7.9	9.5	7.5
	3000	6.5	9.6	6.4	9.7
Small intestine	10	2.3	−1.1	2.7	−1.5
	200	3.2	4.3	3.8	4.9
	3000	5.1	2.9	5.5	2.3
Bladder	10	3.5	7.6	3.5	7.1
	200	4.2	1.1	4.7	1.7
	3000	3.5	−0.3	3.4	−0.5
Brain	10	5.4	2.1	5.1	1.8
	200	3.9	7.5	3.7	6.9
	3000	7.5	2.4	7.6	2.3
Ileum	10	3.3	5.5	3.8	5.2
	200	4.3	6.7	4.5	7.1
	3000	3.3	4.4	3.1	4.3
Cecum	10	5.5	7.6	5.4	7.9
	200	3.2	5.3	3.1	5.1
	3000	4.9	6.7	5.3	7.1
Jejunum	10	3.3	4.9	3.1	5.1
	200	5.5	1.8	5.1	2.1
	3000	5.7	3.5	4.9	3.2
Colon	10	1.8	2.5	2.1	2.9
	200	1.4	−1.5	1.2	−1.8
	3000	2.1	1.7	1.9	1.9
Urinary	10	3.8	2.1	3.6	1.9
	200	5.4	3.9	5.7	3.5
	3000	3.6	5.4	3.3	5.1
Fecal	10	4.1	6.3	3.2	4.6
	200	2.9	4.6	2.5	3.8
	3000	3.3	5.1	3.5	4.3

**Table 3:** Extraction recovery and matrix effect obtained for DPTM analysis in plasma, tissue homogenates, urine and feces (*n* = 6).

Biosamples	Spiked Concentration (ng/mL)	Extraction Recovery		Matrix Effect	
		Mean ± SD (%)	RSD (%)	Mean ± SD (%)	RSD (%)
Plasma	10	101 ± 6	7.6	103 ± 3	6.9
	200	103 ± 4	3.5	103 ± 6	7.3
	3000	100 ± 3	8.9	101 ± 4	6.5
IS	2000	101 ± 5	3.4	98 ± 6	7.3
Heart	10	99 ± 3	9.8	97 ± 6	8.6
	200	103 ± 5	7.6	99 ± 7	5.4
	3000	100 ± 7	9.3	99 ± 7	3.6
IS	2000	97 ± 4	8.6	94 ± 7	5.3
Liver	10	99 ± 6	5.6	97 ± 6	7.8
	200	103 ± 7	5.8	99 ± 5	2.1
	3000	101 ± 5	6.3	99 ± 3	2.5
IS	2000	99 ± 2	7.9	100 ± 7	1.2
Spleen	10	95 ± 4	8.6	95 ± 3	6.5
	200	97 ± 4	7.9	99 ± 6	7.3
	3000	103 ± 8	9.8	99 ± 7	6.5
IS	2000	101 ± 7	7.8	98 ± 4	7.3
Lung	10	100 ± 7	6.2	99 ± 7	6.5
	200	98 ± 4	7.5	97 ± 6	3.8
	3000	109 ± 6	9.8	98 ± 9	4.2
IS	2000	103 ± 4	7.3	99 ± 9	5.1
Kidney	10	105 ± 6	3.2	99 ± 7	2.3
	200	100 ± 5	2.6	102 ± 4	3.2
	3000	99 ± 6	3.8	102 ± 8	5.4
IS	2000	99 ± 2	6.1	104 ± 7	6.9
Large intestine	10	107 ± 6	9.3	104 ± 7	7.2
	200	103 ± 4	7.9	104 ± 6	6.7
	3000	107 ± 9	7.9	105 ± 6	3.2
IS	2000	103 ± 4	7.6	106 ± 9	2.3
Small intestine	10	100 ± 6	6.5	99 ± 8	5.1
	200	109 ± 4	7.3	100 ± 4	3.2
	3000	99 ± 6	3.2	98 ± 8	7.6
IS	2000	99 ± 8	2.1	99 ± 4	8.5
Bladder	10	97 ± 4	3.5	98 ± 7	6.8
	200	100 ± 8	6.3	99 ± 7	5.4
	3000	99 ± 7	5.9	97 ± 10	8.3
IS	2000	109 ± 8	9.5	97 ± 4	5.8
Brain	10	110 ± 8	8.1	107 ± 5	3.8
	200	107 ± 4	4.5	101 ± 5	6.2
	3000	103 ± 4	6.8	105 ± 7	5.8
IS	2000	103 ± 5	7.8	110 ± 5	5.5
Ileum	10	109 ± 8	6.6	105 ± 8	4.5
	200	103 ± 6	4.9	102 ± 5	6.8
	3000	98 ± 7	2.4	103 ± 3	4.3
IS	2000	101 ± 8	3.2	101 ± 6	3.8
Cecum	10	103 ± 6	2.8	106 ± 8	4.5
	200	101 ± 7	4.5	102 ± 4	5.6
	3000	103 ± 6	5.1	104 ± 4	4.5
IS	2000	109 ± 7	3.6	106 ± 5	6.5
Jejunum	10	101 ± 6	5.5	107 ± 5	7.5
	200	104 ± 3	7.4	99 ± 8	6.4
	3000	99 ± 4	8.6	97 ± 9	6.9
IS	2000	102 ± 10	9.2	104 ± 6	5.8
Colon	10	97 ± 9	8.3	108 ±3	5.5
	200	94 ± 7	7.4	101 ±8	5.4
	3000	106 ± 8	9.9	104 ± 10	6.7
IS	2000	98 ± 7	6.3	107 ± 7	8.2
Urinary	10	101 ± 9	6.2	102 ± 8	9.1
	200	99 ± 7	6.7	106 ± 6	6.8
	3000	104 ± 7	5.9	109 ± 4	7.4
IS	2000	103 ± 7	6.4	101 ± 8	6.8
fecal	10	101 ± 6	7.2	102 ± 9	9.3
	200	107 ± 3	6.8	105 ± 2	9.4
	3000	102 ± 10	9.1	106 ± 9	8.6
IS	2000	98 ± 9	7.7	102 ± 7	5.4

**Table 4:** Stability of DPTM in plasma, tissue homogenates, urine and feces.

Biosamples	Analyte Concentration (ng/mL)	Short-term (25 °C, 24 h)		Freeze-thaw Cycles		Long-term (−70 °C, 4 Weeks)	
		RE (%)	RSD (%)	RE (%)	RSD (%)	RE (%)	RSD (%)
Plasma	10	5.1	7.9	6.3	7.7	5.8	7.8
	200	6.8	7.6	7.9	4.1	7.3	8.4
	3000	1.5	5.4	1.7	5.4	2	5.1
Heart	10	3.9	4.3	3.5	8.7	3.3	8
	200	−0.7	2.8	−0.3	0.5	−0.7	0.3
	3000	6.3	7.1	6.3	4.2	6.5	4.6
Liver	10	2.9	5.1	3.3	6.5	3.4	6.3
	200	3.3	4.7	3.4	5.6	3.6	5.4
	3000	6.1	5.3	1.9	2.2	2.1	3.8
Spleen	10	3.7	1.4	3.6	4.7	3.3	4.9
	200	7.2	−1.8	7.9	−1.5	7.1	5.2
	3000	5.9	7.1	5.6	3.5	5.7	3.6
Lung	10	3.3	4.6	2.5	2.7	2.9	4.4
	200	2.1	2.9	3.1	5.2	3.5	5.4
	3000	1.3	1.1	7.6	9.4	7.5	9.6
Kidney	10	3.4	5.1	3.7	4.5	3.5	4.3
	200	0.9	2.1	2.3	3.3	1.9	2.7
	3000	3.2	6.3	10.1	7.6	9.9	7.3
Large intestine	10	1.9	3.1	6.3	9.3	6.9	10.2
	200	5.7	5.4	2.4	4	2.2	7.1
	3000	−0.9	4.1	3.1	4.1	3.5	4.5
Small intestine	10	1.3	3.1	4.9	3.1	4.6	3.2
	200	3.7	1.5	3.2	7.3	3.6	7.4
	3000	5.3	3.2	3.9	1.3	3.8	1.2
Bladder	10	4.1	6.3	3.9	2.1	3.6	1.7
	200	1.9	3.4	5.1	2	5.1	1.9
	3000	3.1	1.8	3.5	7.1	3.3	6.9
Brain	10	1.1	5.1	4.9	7.3	5.3	7.3
	200	3.3	4.5	6.5	5.1	6.6	9.3
	3000	8.1	5.5	4.1	6.5	4.1	6.2
Ileum	10	2.9	4.1	−3.5	4.1	3.2	4.5
	200	1.7	1.2	5.2	7.3	−5.1	7.1
	3000	3.1	4.5	3.3	5.1	3.5	4.9
Cecum	10	6.7	5.5	7.2	5.5	6.4	2.1
	200	3.1	7.2	3.9	6.3	3.2	7.5
	3000	−5.1	4.8	−4.9	4.5	−6.4	3.8
Jejunum	10	7.2	3.6	6.1	7.2	5.6	7.2
	200	7.5	4.8	7.3	4.9	7.3	4.8
	3000	−5.3	4.6	−5.4	4.8	−5.6	5.1
Colon	10	5.4	3.2	6.3	4.5	4.8	3.7
	200	6.8	5.9	7.2	8.9	2.2	5.4
	3000	7.3	8.4	−4.3	6.5	2.3	5.5
Urinary	10	6.6	2.1	8.1	7.2	8.4	6.6
	200	5.3	4.5	4.2	3.4	−1.2	5.9
	3000	−5.6	7.2	−5.5	6.8	−3.3	4.5
Fecal	10	3.2	5.7	4.8	3.6	3.3	3.9
	200	2.9	4.1	3.6	3.9	4.1	3.7
	3000	4.8	5.1	4.7	4.5	4.3	4.6

**Table 5:** Pharmacokinetic parameters of DPTM in serum after i.v., i.m. and p.o. administration.

Parameters	i.v.			i.m.			p.o.		
	10	25	75	10	25	75	10	25	75
*C*_max_ (μg/mL) *	7.5 ± 0.4	16.0 ± 0.5	65.1 ± 0.8	0.5 ± 0.1	0.7 ± 0.1	1.8 ± 0.1	0.1 ± 0.0	0.2 ± 0.0	0.3 ± 0.1
*T*_max_ (h)	0.08 (0.08–1.00)	0.08 (0.08–1.00)	0.083 (0.08–1.00)	0.50 (0.17–4.00)	0.50 (0.17–4.00)	0.50 (0.17–4.00)	2.00 (0.17–6.00)	2.00 (0.17–6.00)	2.00 (0.17–6.00)
*t*_1/2_ (h)	1.7 ± 0.1	1.7 ± 0.4	1.9 ± 0.5	3.5 ± 0.0	3.2 ± 0.2	3.3 ± 0.3	4.6 ± 0.1	4.4 ± 0.2	4.7 ± 0.4
CL (L/h/kg)*	0.3 ± 0.1	0.1 ± 0.0	0.03 ± 0.0	–	–	–	–	–	–
MRT_0–t_ (h)	1.0 ± 0.2	1.3 ± 0.7	1.5 ± 0.4	4.4 ± 0.3	4.8 ± 0.3	4.2 ± 0.8	12.8 ± 0.1	9.8 ± 0.2	6.9 ± 0.4
*V*_ss_ (L/kg)*	0.3 ± 0.1	0.2 ± 0.0	0.03 ± 0.0	–	–	–	–	–	–
AUC_0→t_ (μg·h/mL)*	2.2 ± 0.2	5.4 ± 0.2	36.0 ± 0.3	1.5 ± 0.1	2.6 ± 0.2	5.7 ± 0.2	0.5 ± 0.1	1.3 ± 0.1	1.5 ± 0.0
CL_R(__0–t__)_ (mL/min)	–	–	97.6 ± 9.6	–	–	46.2 ± 3.8	–	–	12.2 ± 1.3
*F* (%)	–	–	–	68.16	48.52	15.84	22.87	24.26	4.17

*, parameters with dose-dependent; *C*_max_, maximum serum concentration; *t_max_*, time of maximum serum concentration; *t*_1/2_, elimination half-life; CL, clearance; MRT_0–t_, mean resident time; *V*_ss_, distribution volume at steady state; AUC_0→t_, area under serum concentration-time curve; CL_R(0–t)_ renal clearance of ; *F*, absolute bioavailability.

## Supplementary Materials

Supplementary materials are available on line.
