# Supplementary material for: A Validated HPLC-MS/MS Assay for 14-O-[(4,6-Diaminopyrimidine-2-yl)thioacetyl] Mutilin in Biological Samples and Its Pharmacokinetic, Distribution and Excretion via Urine and Feces in Rats
Source: Molecules. 2019 Feb 22;24(4):790. doi: 10.3390/molecules24040790 (PMC6413085; doi:10.3390/molecules24040790)
Supplement: Supplementary file 1 [file molecules-24-00790-s001.pdf]

A Validated HPLC-MS/MS Assay for 14-*O*-[(4,6-Diaminopyrimidine-2-yl)thioacetyl] Mutilin in Biological Samples and Its Pharmacokinetic, Distribution and Excretion via Urine and Feces in Rats

Yunxing Fu [1], Yu Liu [1], Yunpeng Yi [1], Jianping Liang [1], Qingfeng Wu [2,*] and Ruofeng Shang [1,*]

[1] Key Laboratory of New Animal Drug Project of Gansu Province, Key Laboratory of Veterinary Pharmaceutical Development, Ministry of Agriculture; Lanzhou Institute of Husbandry and Pharmaceutical Sciences of CAAS, Lanzhou 730050, China; fyx1261648623@163.com (Y.F.); yangguang8684@163.com (Y.L); yiyp@foxmail.com (Y.Y.); liangjp100@sina.com (J.L.)

[2] Institute of Modern Physics, Chinese Academy of Sciences, Lanzhou 730000, China

* Correspondence: wuqf@impcas.ac.cn (Q.W.); shangrf1974@163.com (R.S.); Tel.: +86-931-2115253 (R.S.); Fax: +86-931-2115951 (R.S.)

Supplemental Data

Table of Contents

S1. Typical DPTM chromatograms of blank rat heart 1

S2. Typical DPTM chromatograms of blank rat liver 1

S3. Typical DPTM chromatograms of blank rat spleen 2

S4. Typical DPTM chromatograms of blank rat lung 2

S5. Typical DPTM chromatograms of blank rat kidney 3

S6. Typical DPTM chromatograms of blank rat large intestine 3

S7. Typical DPTM chromatograms of blank rat small intestine 4

S8. Typical DPTM chromatograms of blank rat ileum 4

S9. Typical DPTM chromatograms of blank rat cecum 5

S10. Typical DPTM chromatograms of blank rat bladder 5

S11. Typical DPTM chromatograms of blank rat brain 6

S12. Typical DPTM chromatograms of blank rat jejunum 6

S13. Typical DPTM chromatograms of blank rat colon 7

S12. Typical DPTM chromatograms of blank rat urine 7

S13. Typical DPTM chromatograms of blank rat feces 8

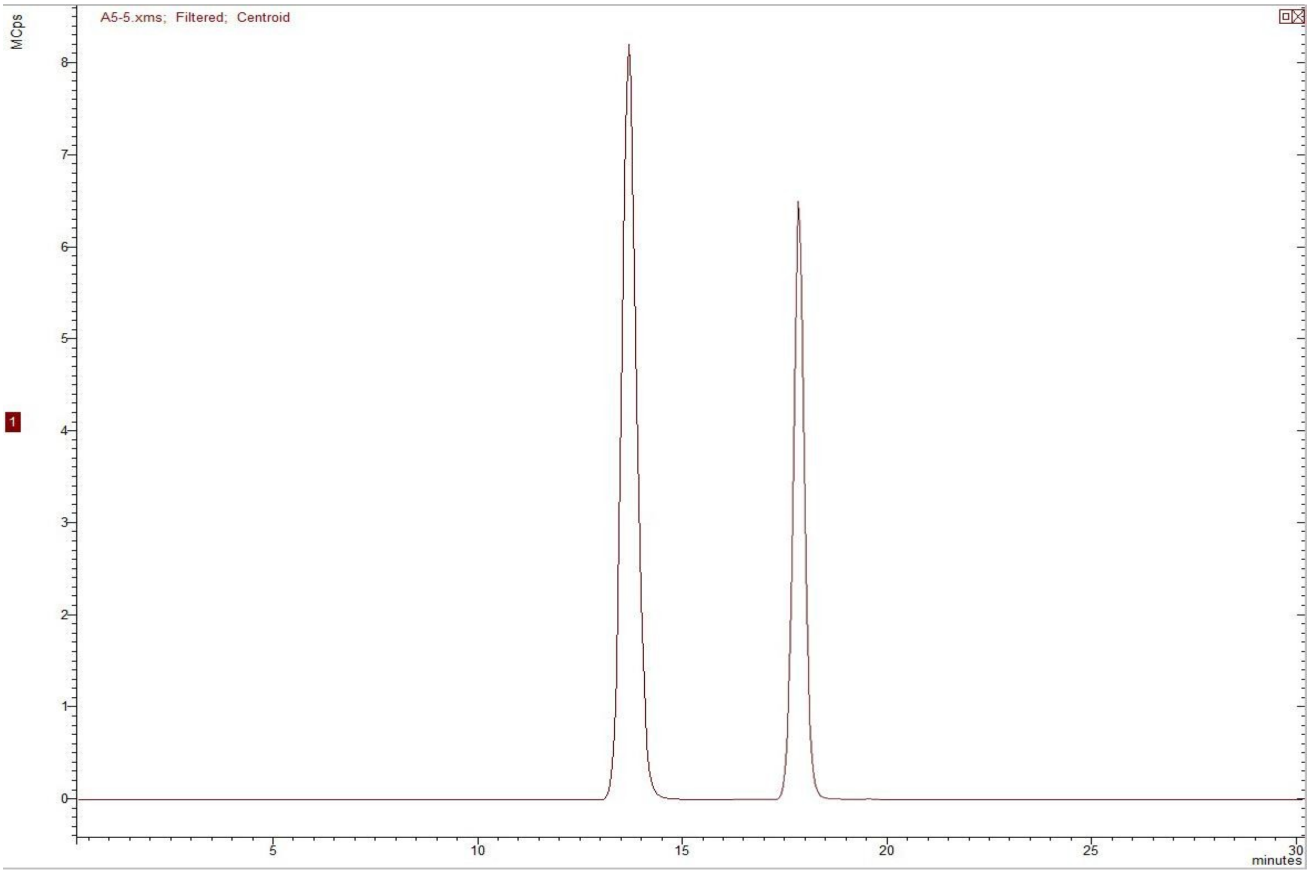

S1. Typical DPTM chromatograms of blank rat heart.

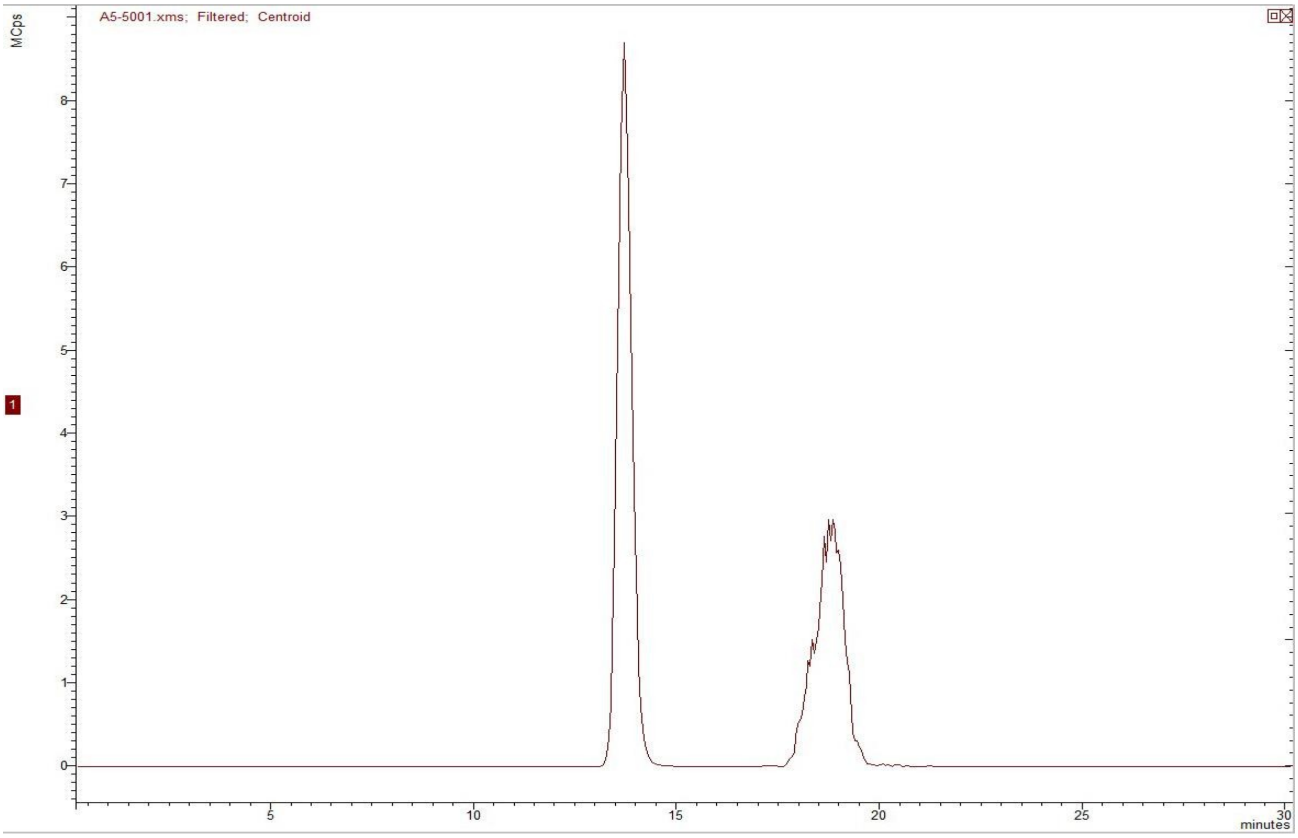

S2. Typical DPTM chromatograms of blank rat liver.

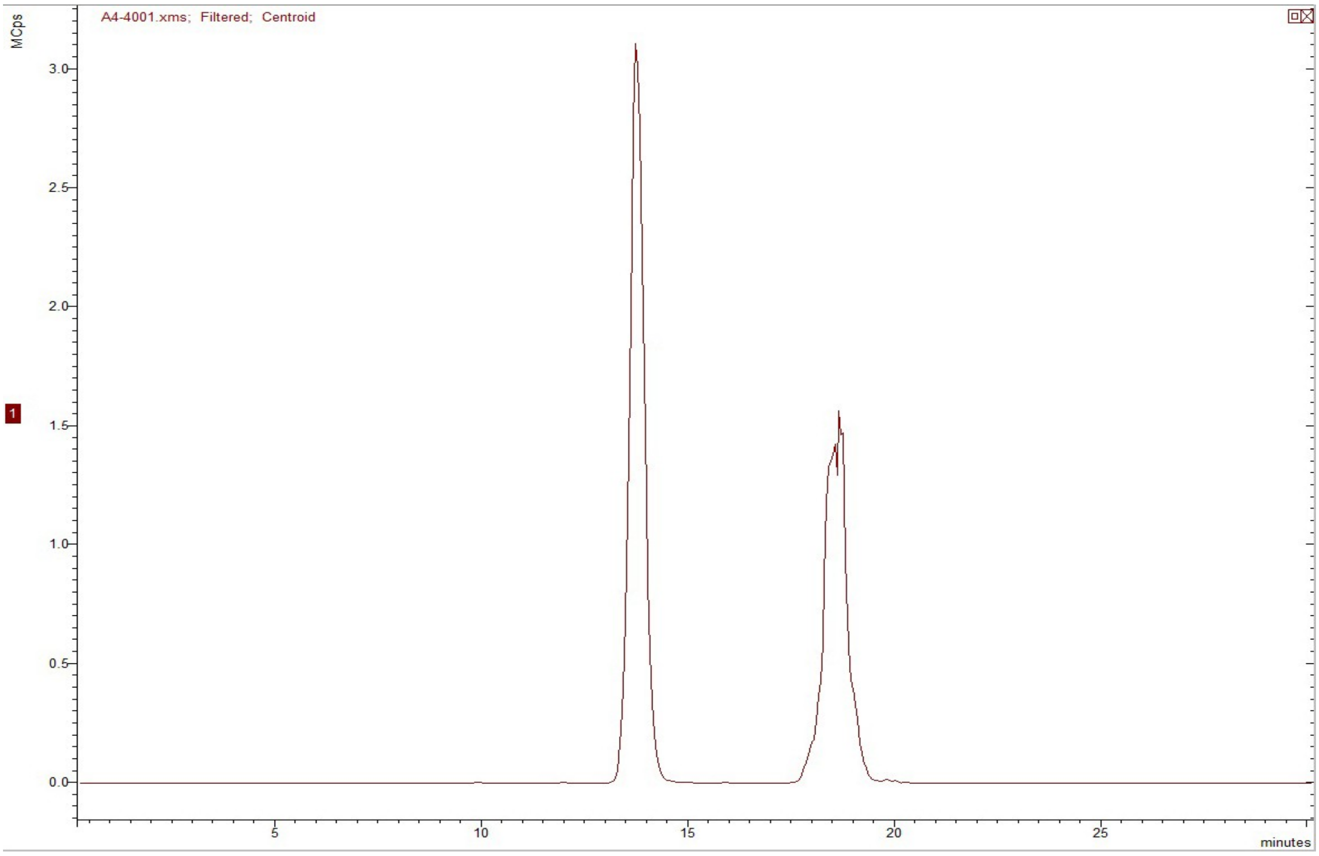

S3. Typical DPTM chromatograms of blank rat spleen.

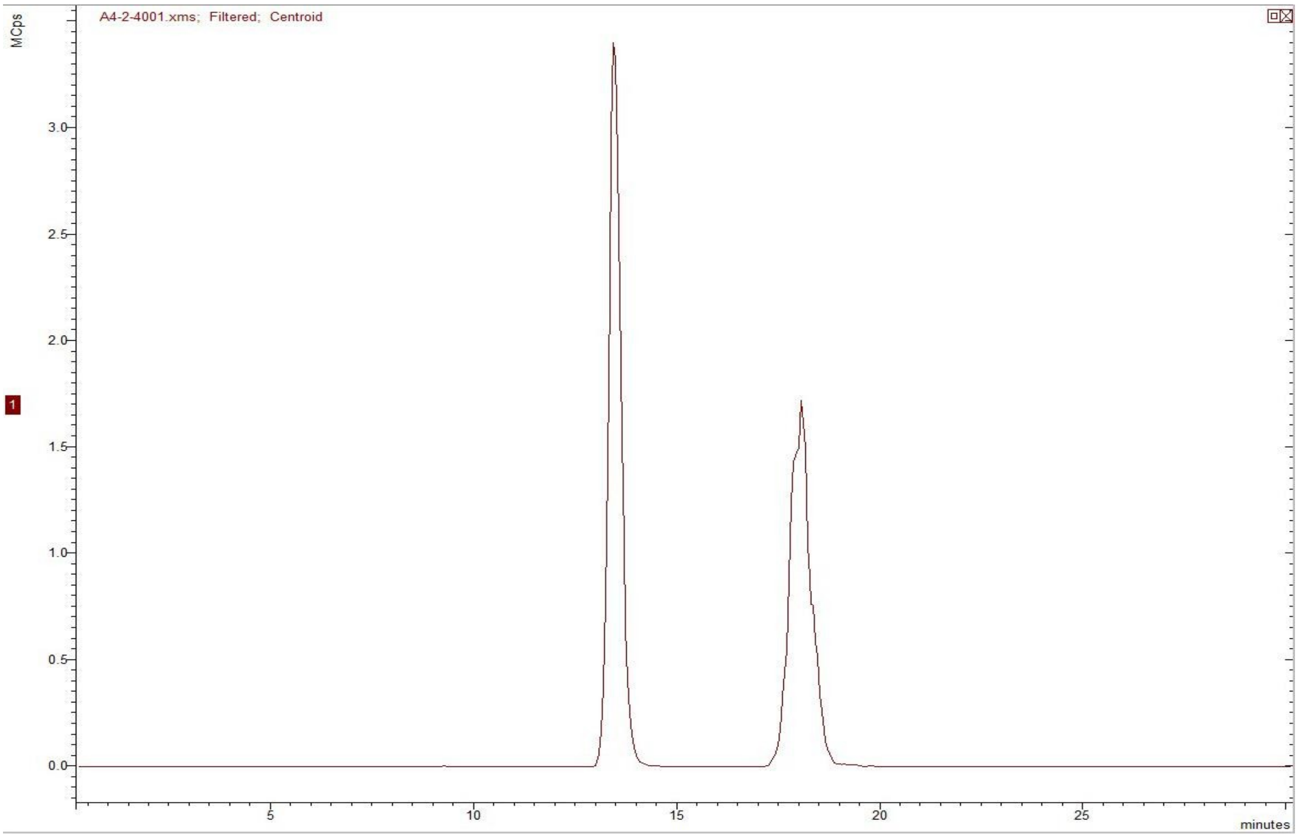

S4. Typical DPTM chromatograms of blank rat lung.

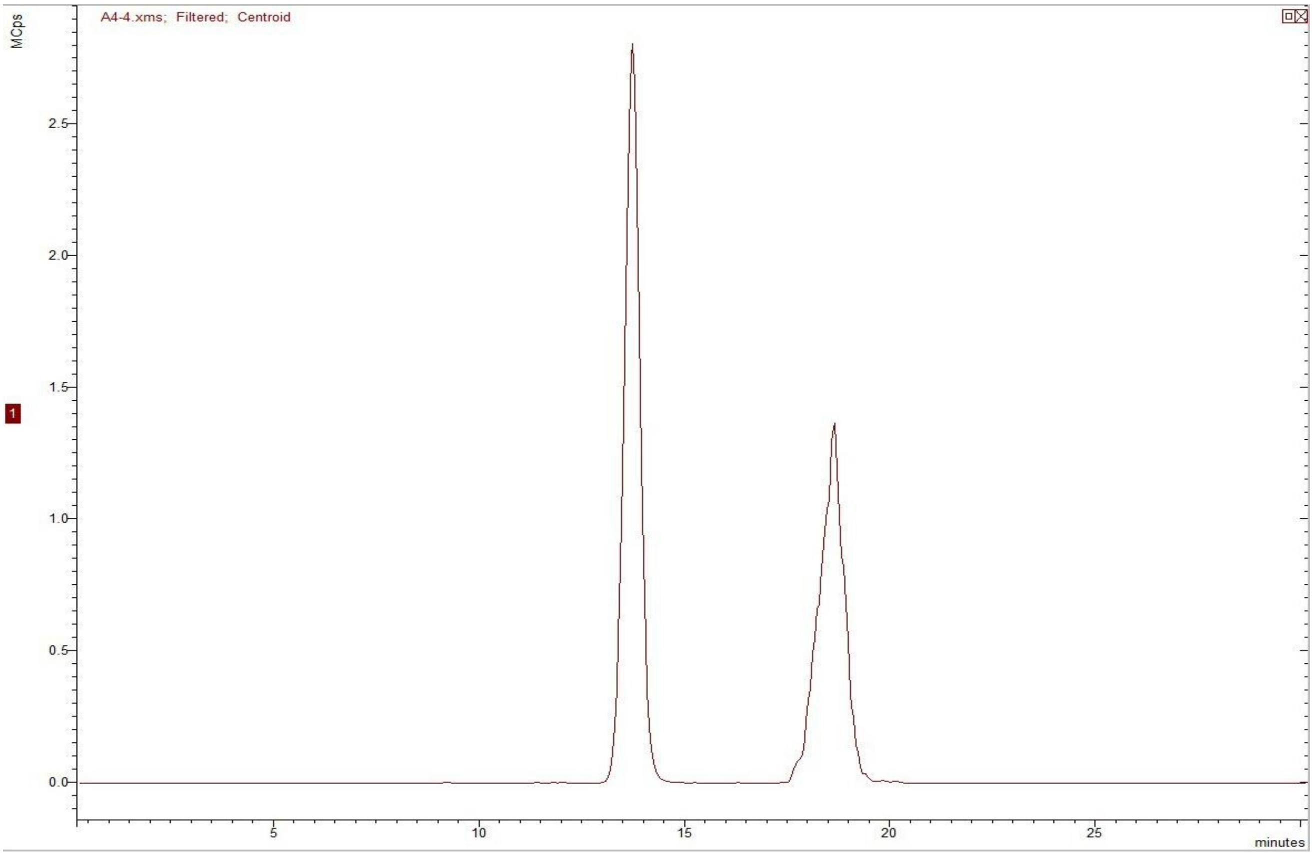

S5. Typical DPTM chromatograms of blank rat kidney.

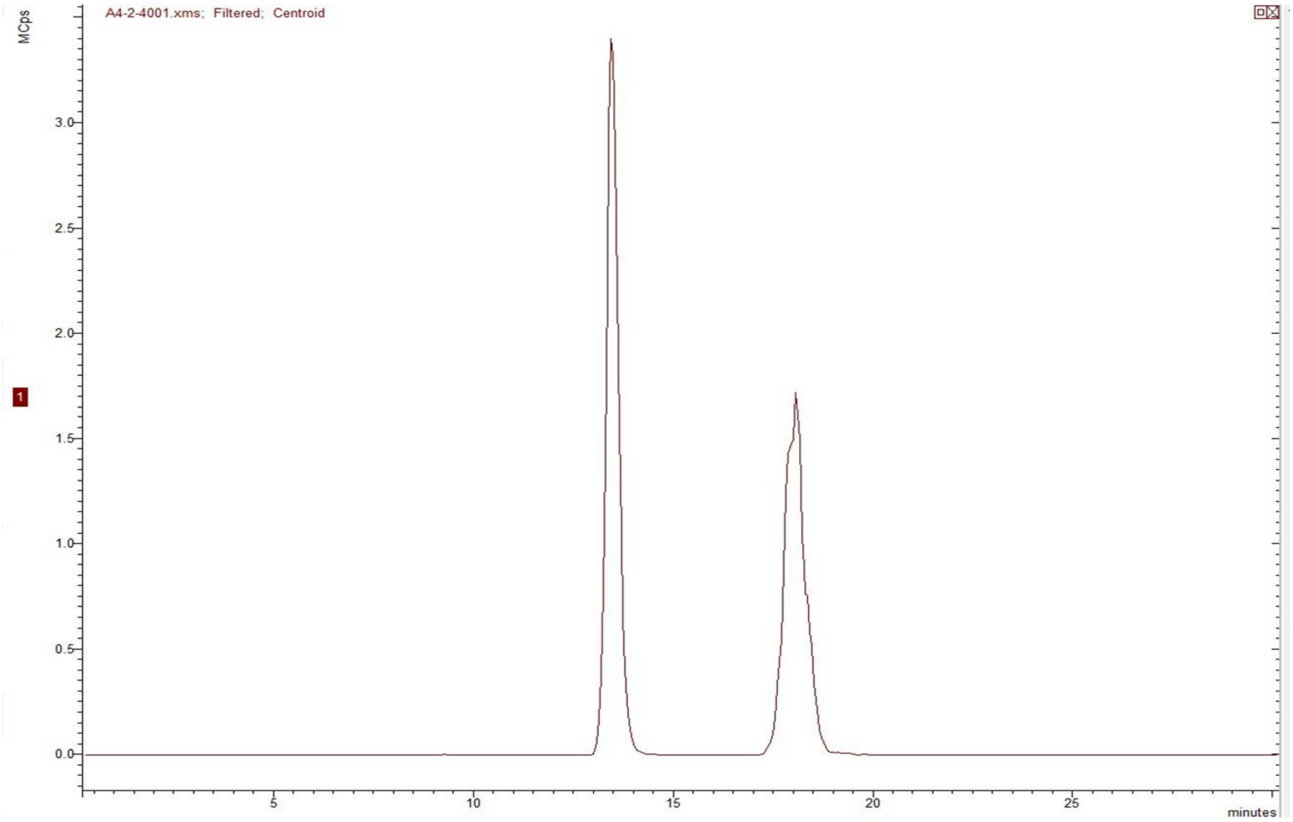

S6. Typical DPTM chromatograms of blank rat large intestine.

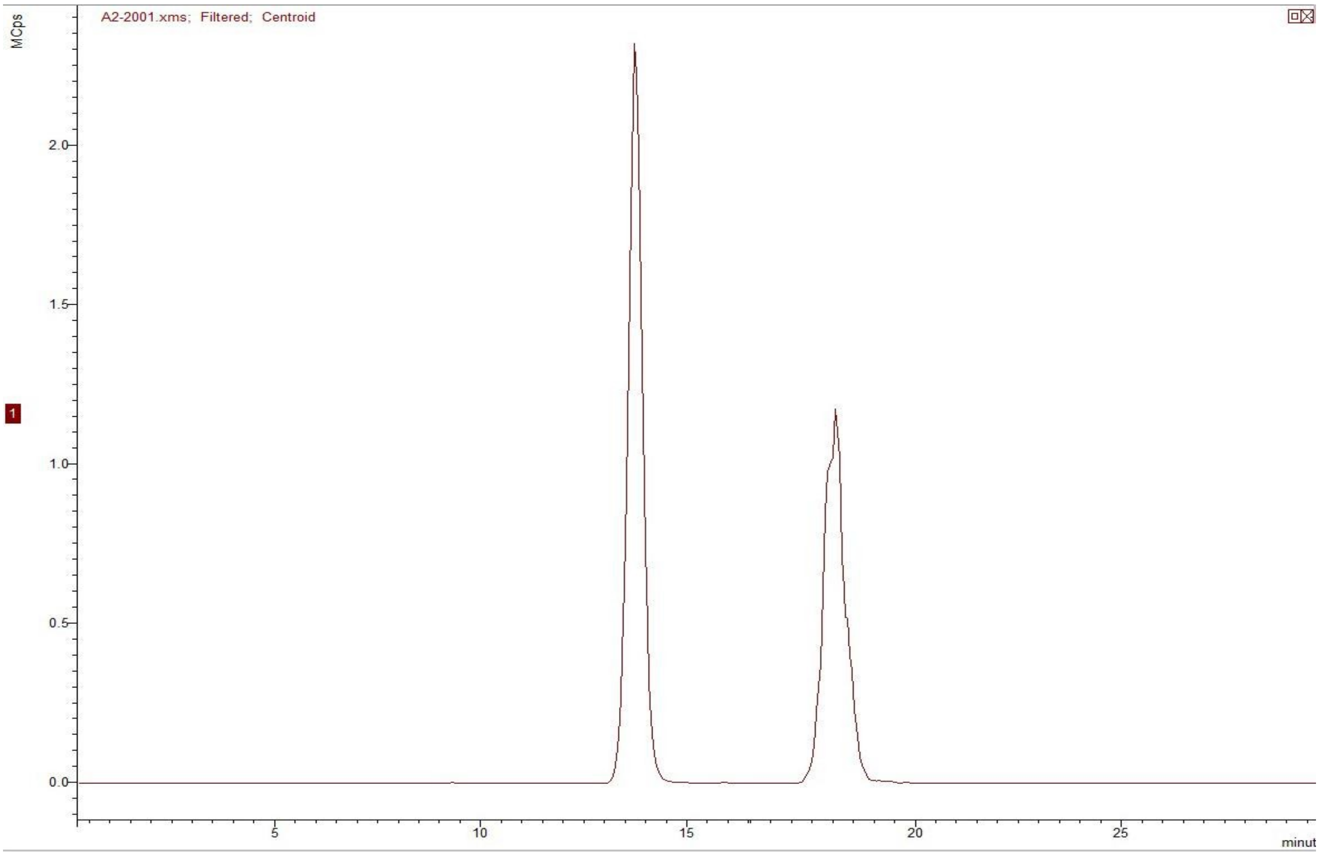

S7. Typical DPTM chromatograms of blank rat small intestine.

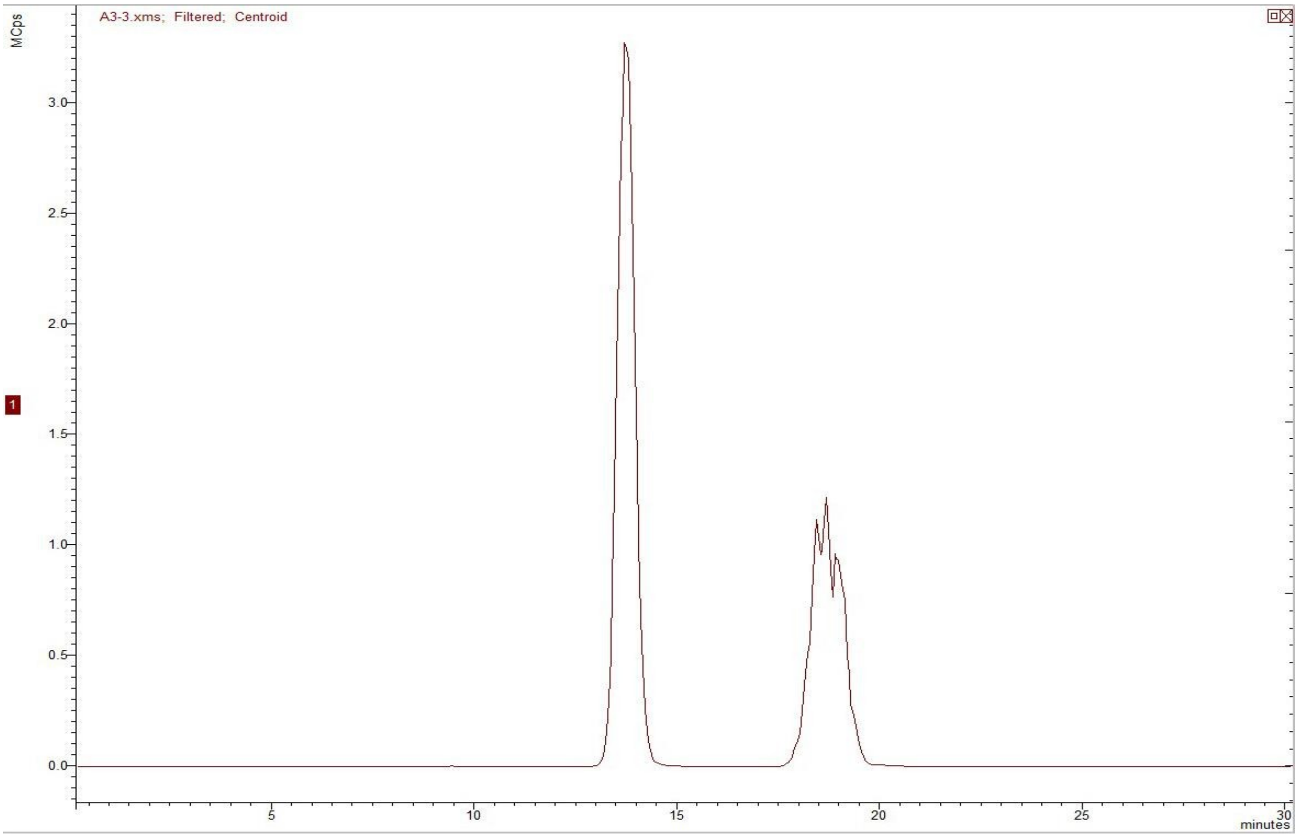

S8. Typical DPTM chromatograms of blank rat ileum.

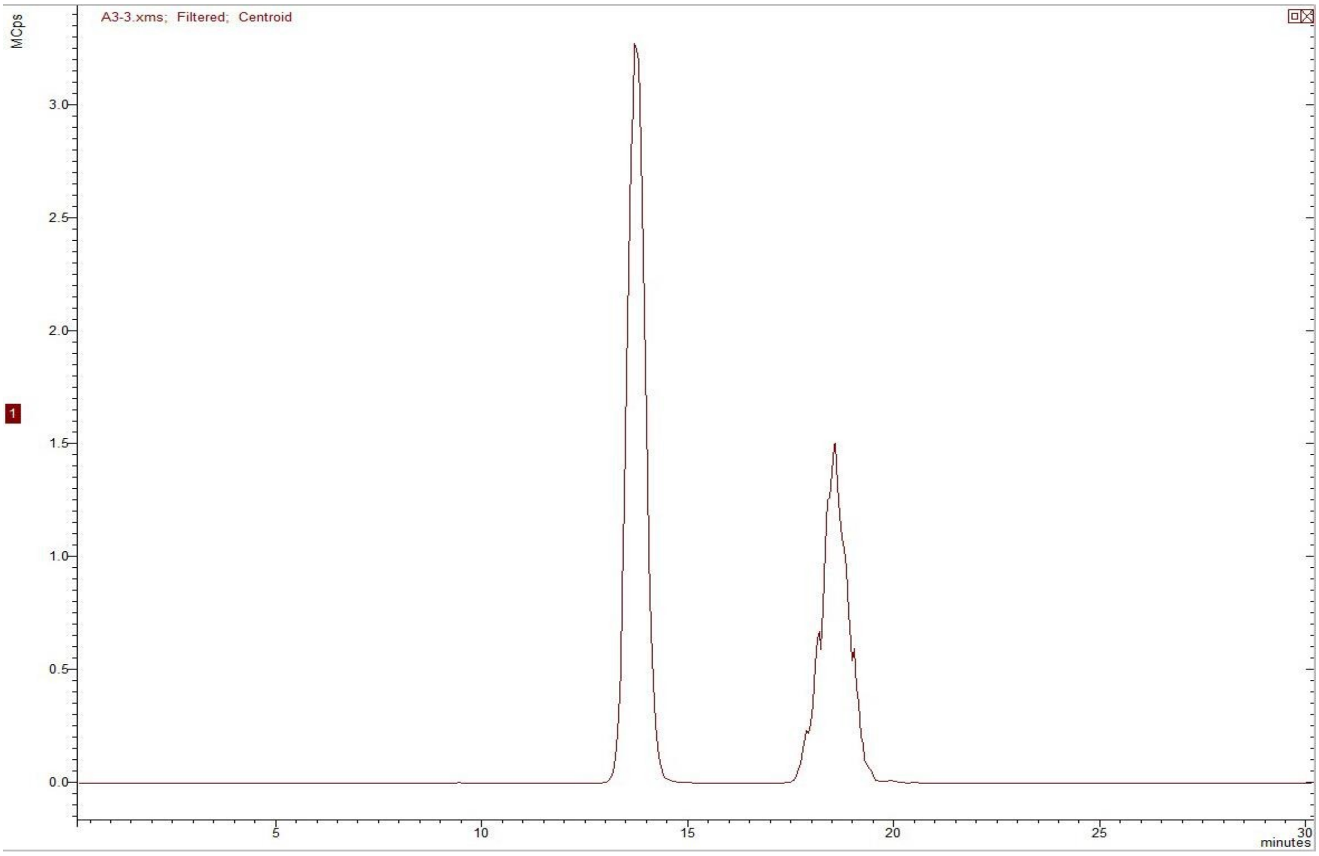

S9. Typical DPTM chromatograms of blank rat cecum.

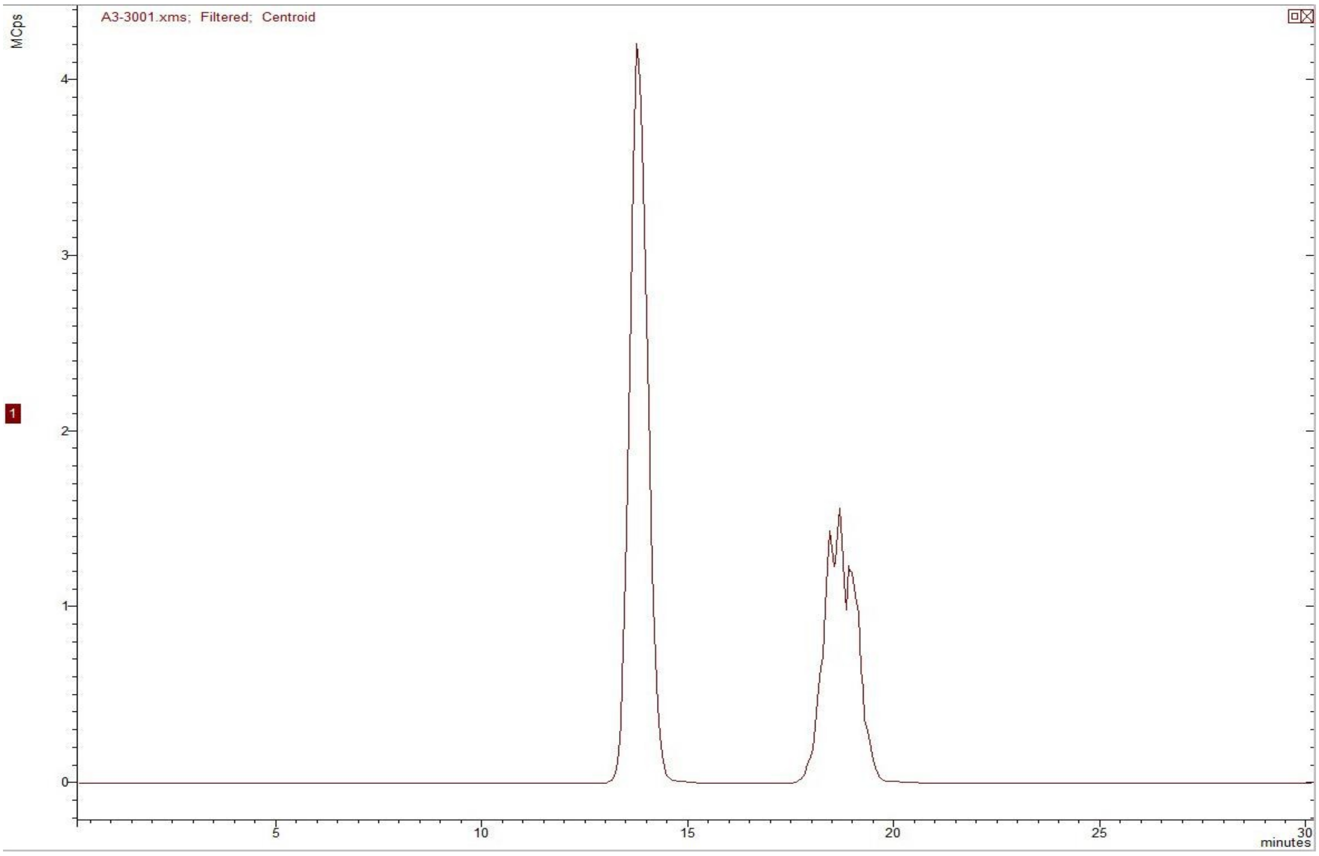

S10. Typical DPTM chromatograms of blank rat bladder.

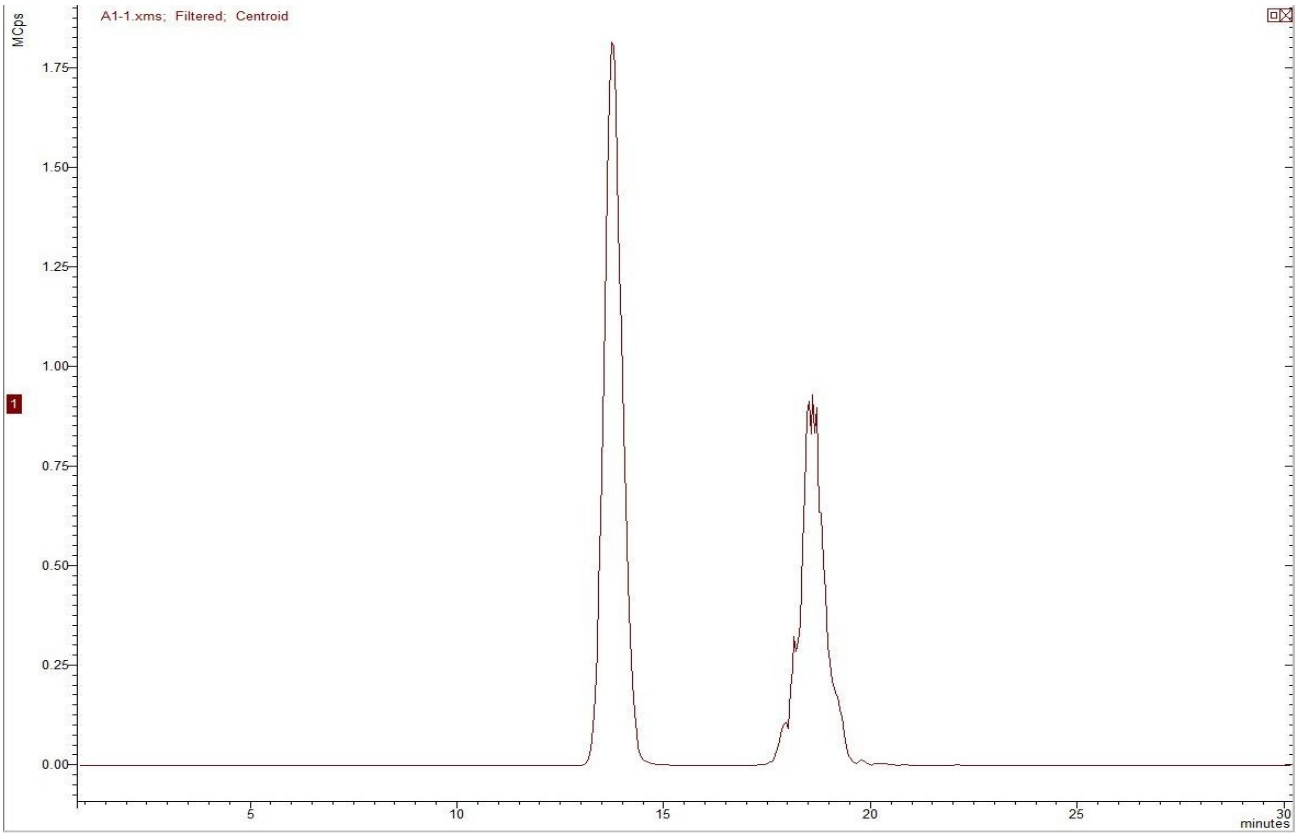

S11. Typical DPTM chromatograms of blank rat brain.

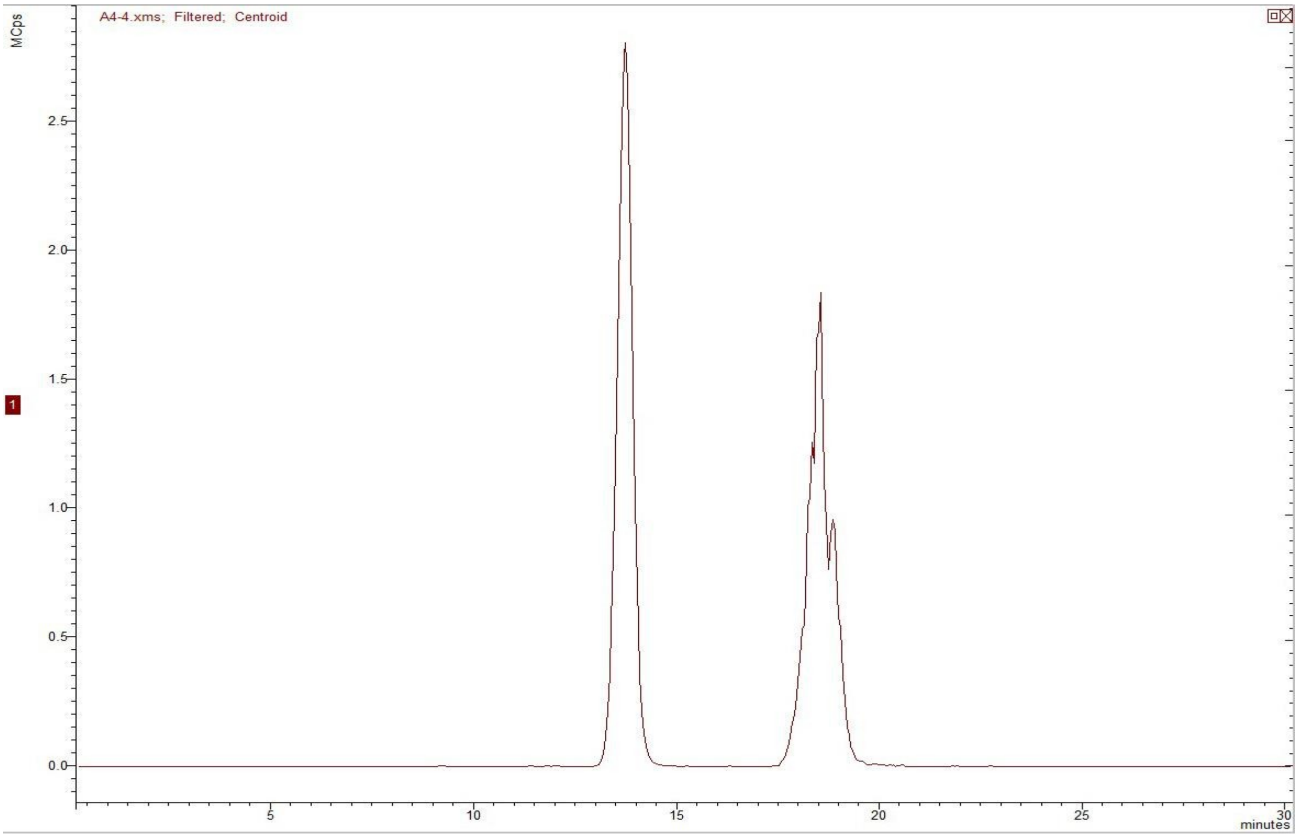

S12. Typical DPTM chromatograms of blank rat jejunum.

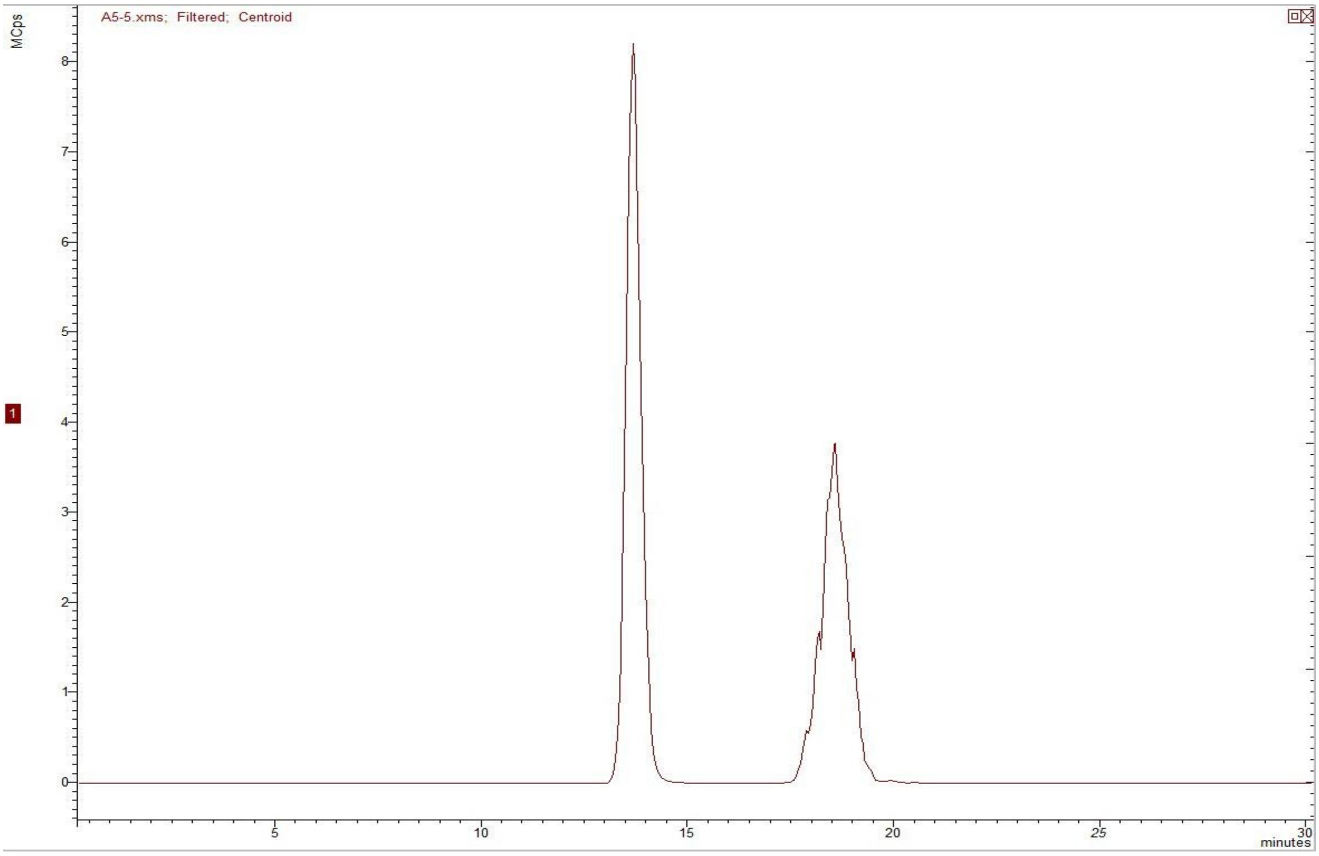

S13. Typical DPTM chromatograms of blank rat colon.

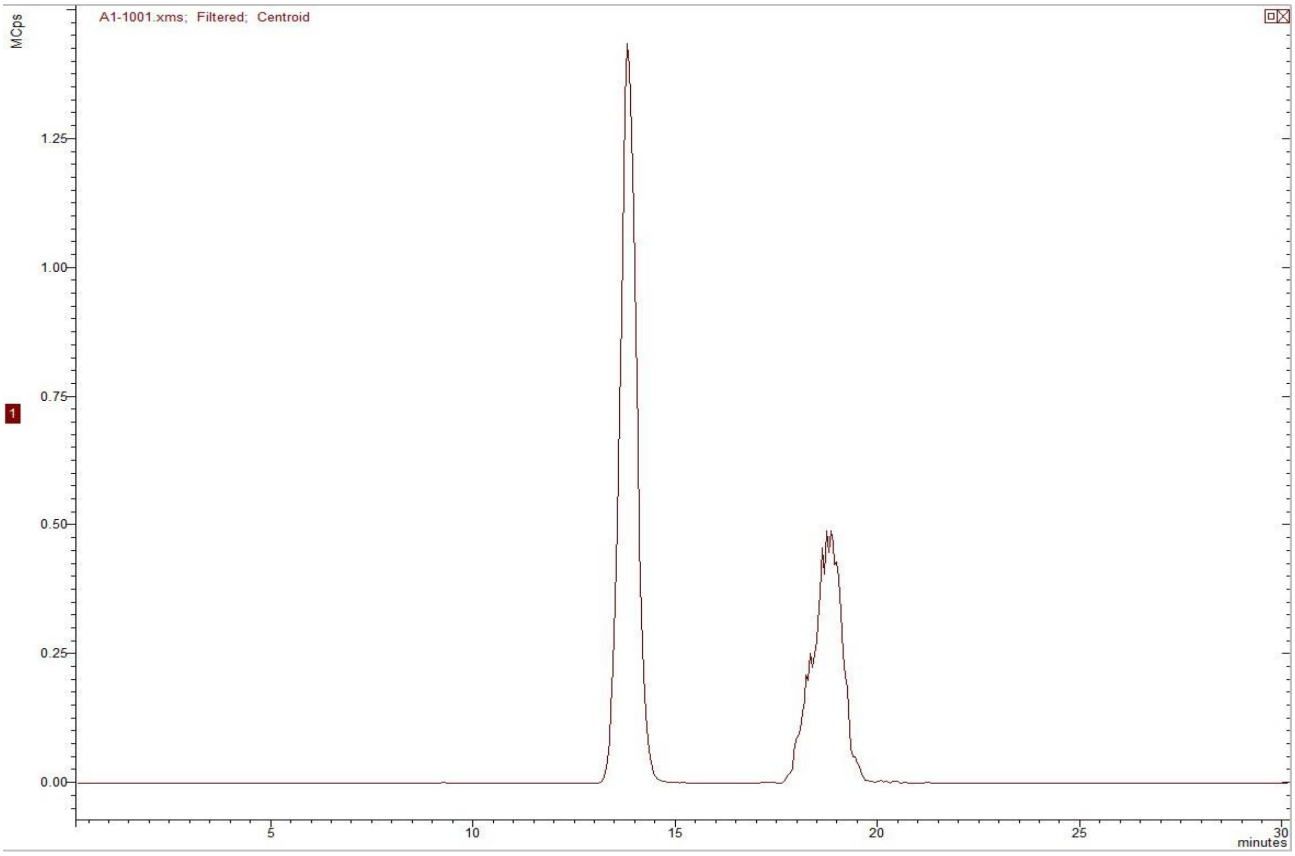

S14. Typical DPTM chromatograms of blank rat feces.

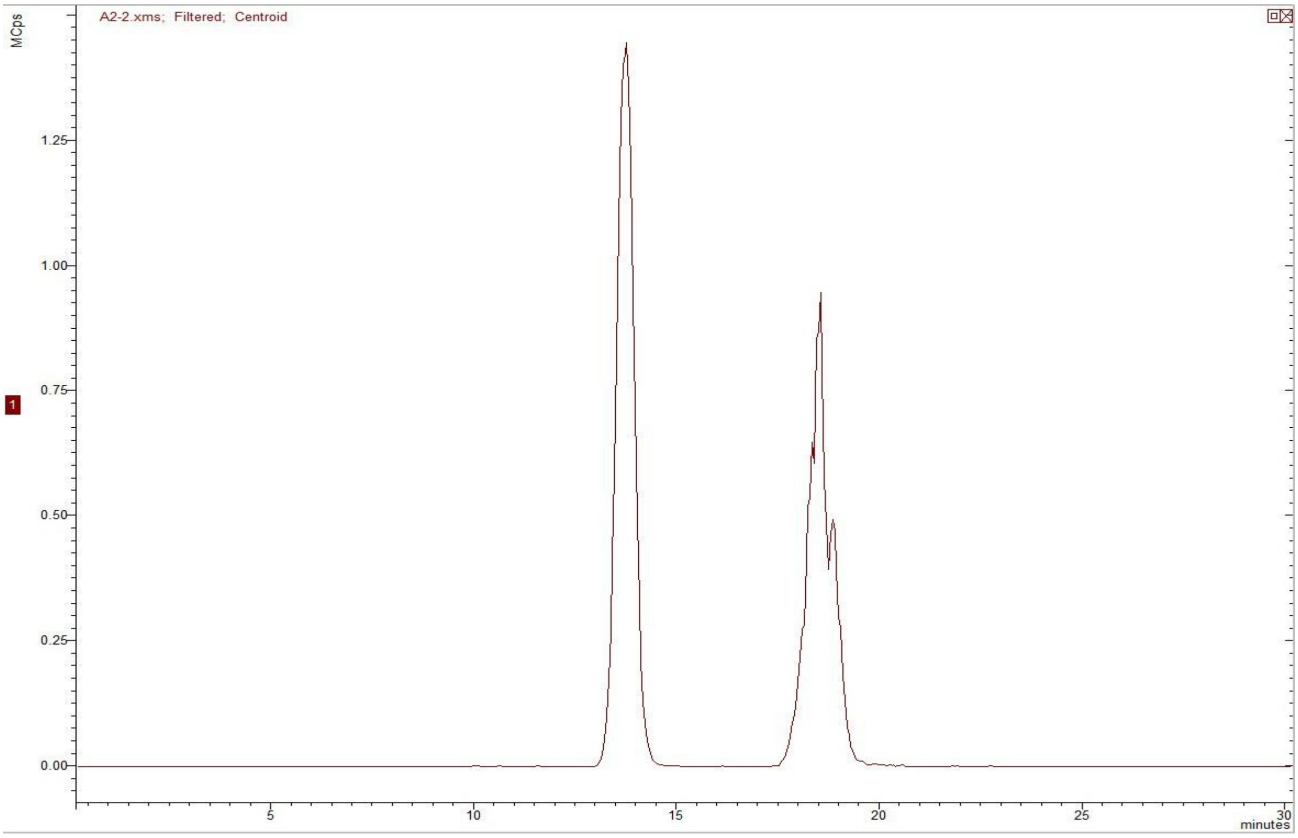

S15. Typical DPTM chromatograms of blank rat urine.
